# Tracing the expression of circular RNAs in human pre-implantation embryos

**DOI:** 10.1186/s13059-016-0991-3

**Published:** 2016-06-17

**Authors:** Yujiao Dang, Liying Yan, Boqiang Hu, Xiaoying Fan, Yixin Ren, Rong Li, Ying Lian, Jie Yan, Qingqing Li, Yan Zhang, Min Li, Xiulian Ren, Jin Huang, Yuqi Wu, Ping Liu, Lu Wen, Chen Zhang, Yanyi Huang, Fuchou Tang, Jie Qiao

**Affiliations:** Biodynamic Optical Imaging Center & Department of Obstetrics and Gynecology, College of Life Sciences, Third Hospital, Peking University, Beijing, 100871 China; Key Laboratory of Assisted Reproduction, Ministry of Education, Beijing, 100191 China; Ministry of Education Key Laboratory of Cell Proliferation and Differentiation, Beijing, 100871 China; Peking-Tsinghua Center for Life Sciences, Peking University, Beijing, 100871 China; Center for Molecular and Translational Medicine, Peking University Health Science Center, Beijing, 100191 China; Beijing Key Laboratory of Reproductive Endocrinology and Assisted Reproduction, Beijing, China; College of Engineering, Peking University, Beijing, 100871 China

**Keywords:** SUPeR-seq, circular RNA, human pre-implantation embryos, mRNA quantification, RNA *de novo* assembly

## Abstract

**Background:**

PolyA– RNAs have not been widely analyzed in human pre-implantation embryos due to the scarcity of materials. In particular, circular RNA (circRNA), a novel type of polyA– RNA, has not been characterized during human pre-implantation development.

**Results:**

We systematically analyze polyA+ messenger RNAs (mRNAs) and polyA– RNAs in individual human oocytes and pre-implantation embryos using SUPeR-seq. We *de novo* identify 10,032 circRNAs from 2974 hosting genes. Most of these circRNAs are developmentally stage-specific and dynamically regulated. Many of them are maternally expressed, implying their potentially important regulatory functions in oogenesis and the formation of totipotent zygotes. Comparison between human and mouse embryos reveals both high conservation and clear distinction between these two species. Human pre-implantation embryos generate more types of circRNA compared with mouse embryos and this is associated with a striking increase of the length of the circRNA flanking introns in humans. We also perform RNA *de novo* assembly and identify novel transcript units, many of which are potentially novel long non-coding RNAs.

**Conclusions:**

This study reports the first analysis of the whole transcriptome comprising both polyA+ mRNAs and polyA– RNAs including circRNAs during human pre-implantation development. It provides an invaluable resource for analyzing the unique function and complex regulatory mechanisms of circRNAs during this process.

**Electronic supplementary material:**

The online version of this article (doi:10.1186/s13059-016-0991-3) contains supplementary material, which is available to authorized users.

## Background

The analysis of gene expression dynamics is important to elucidate the molecular mechanisms regulating the developmental processes of human early embryos. We recently analyzed the transcriptome profiles of human pre-implantation embryos at the single-cell level [1]. However, the oligo-d(T) primers used in our previous work only allowed us to detect the polyA+ messenger RNAs (mRNAs), leaving the polyA– RNAs largely unknown.

A specific type of polyA– RNA, circular RNA (circRNA), has recently emerged as a large class of non-coding RNAs in eukaryotic cells [2–4]. The circular transcripts can consist of back-spliced exons [5], introns as ciRNAs [6], or both exons and introns as EIciRNAs [7]. CircRNAs may play important roles—for example, acting as microRNA sponges [8, 9], competing with linear splicing [10], or interacting with the U1 snRNP to regulate gene expression [7]—during several biological processes. The genomic features that promote circRNA biogenesis, such as inverted repeats in the flanking introns [11], longer flanking introns [12], and canonical splicing sites [10], have been investigated in vitro and in vivo. CircRNAs have been identified in many tissues across different species. A recent study of circRNAs in the mammalian brain shows that they are significantly conserved in expression patterns and sequences [13, 14].

To fully reveal a more complete landscape of individual embryo transcriptome, including the newly discovered circRNAs, during human pre-implantation development, a method that can detect both polyA+ mRNAs and polyA– RNAs in a single embryo is needed. However, conventional RNA-sequencing (RNA-seq) methods for polyA– RNAs requires a large amount of starting material and is unsuitable for such scarce and precious samples, and the current single-cell RNA-seq methods are incapable of capturing polyA– RNA species due to the usage of oligo dT as the reverse transcription primers [15–18].

Recently, we have developed a novel single-cell RNA-seq technique, SUPeR-seq [19], which can detect both polyA+ mRNAs and polyA– RNAs from a single mammalian cell. This novel method has been successfully applied for investigating polyA– RNAs including circRNAs during mouse pre-implantation development [19]. Here, we apply SUPeR-seq to systematically analyze the transcriptomes of individual human pre-implantation embryos.

We have identified a total of 10,032 exonic circRNAs from 2974 hosting genes in human pre-implantation embryos, including a large proportion of circRNA hosting genes of mouse pre-implantation embryos. In addition, based on spike-ins, we quantitatively calculated the total copy number of mRNAs in each oocyte or embryo and analyzed the differential expressed genes (DEG) during human pre-implantation development with RPKM normalized by the mRNA content. A total of 5573 maternal genes and 7427 zygotically activated genes during the major wave of the maternal zygotic transition were identified. Based on the DEG analysis, among 2974 circRNA hosting genes, over half of them (1554) were maternal genes and 851 were zygotic genes. This is the first analysis of the whole transcriptome comprised of both polyA+ mRNAs and polyA– RNAs including circRNAs in human pre-implantation embryos.

## Results and discussion

### Global expression dynamics of RefSeq genes during human pre-implantation development

**Fig. 1:**
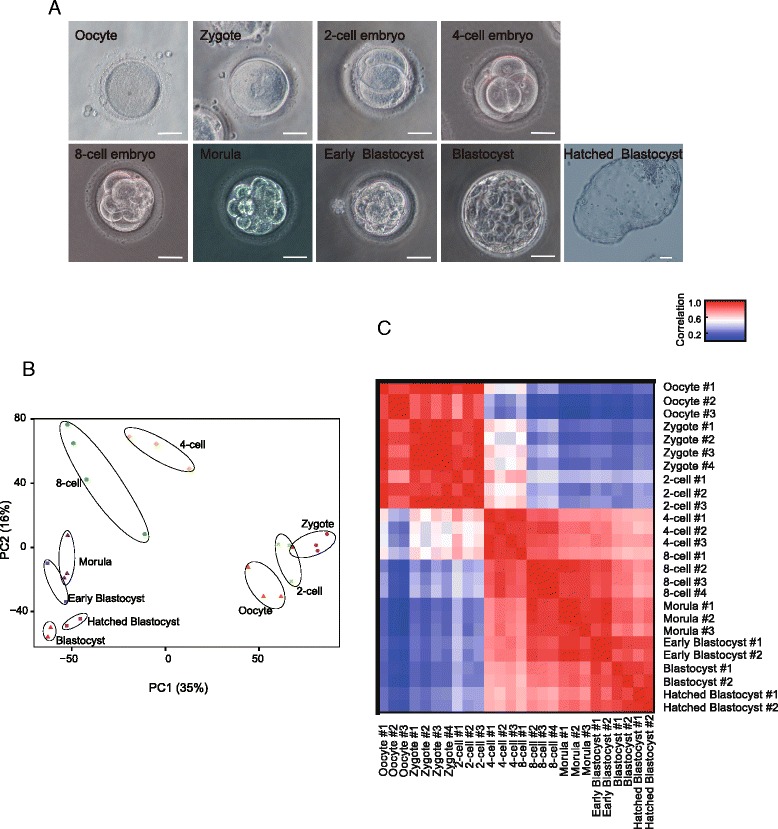
Morphology of human early embryos and global expression pattern of RefSeq genes during human pre-implantation development. **a** *Microscopy images* of human mature oocyte and pre-implantation embryos at zygote (2PN), 2-cell, 4-cell, 8-cell, morula, early blastocyst, blastocyst, and hatched blastocyst stages. Scale bar, 50um. **b** *PCA* of the transcriptome of single embryos during human pre-implantation development. **c** *Pearson correlation coefficient heat map* of single embryo transcriptomes during human pre-implantation development

To determine the expression dynamics of the complete transcriptome, including polyA+ mRNAs and polyA– RNAs, during human pre-implantation development, we sequenced individual cells and embryos at seven consecutive stages (mature oocytes, zygotes, 2-cell, 4-cell, and 8-cell embryos, morulae, and blastocysts) using SUPeR-seq, a single-cell RNA-seq technique we developed recently [19]. Specifically, we profiled the blastocysts at the early blastocyst, blastocyst, and hatched blastocyst stages and analyzed a total of 29 oocytes and embryos (Fig. 1a; Additional file 1: Table S1). Principal component analysis (PCA) showed that the embryos at the same developmental stage clustered together properly and the embryos at different stages separated from each other as expected (Fig. 1b). The analysis of global Pearson correlation coefficients showed a similar pattern (Fig. 1c). In addition, the correlation coefficients between replicates at the same stage are high (Additional file 2: Figure S1A, in the red box). To further verify the stability of the SUPeR-seq method, we also compared the External RNA Controls Consortium (ERCC) RNAs spiked in these samples. The average Pearson correlation coefficients of ERCC between different samples was 0.94, verifying that the SUPeR-seq method is reproducible for measuring gene expression of individual human pre-implantation embryos (Additional file 2: Figure S1B). The proportion of duplicated pair-end reads in our SUPeR-seq data is low (Additional file 2: Figure S1C).

**Fig. 2:**
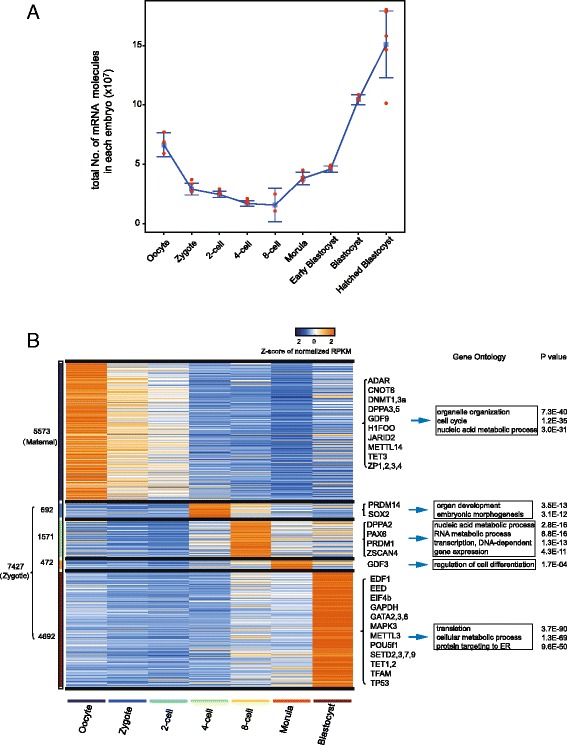
mRNA quantification and analysis of differential expressed genes. **a** mRNA copy numbers in each individual embryo during human pre-implantation development were estimated by the algorithm of ERCC/RGC-A80 normalization. The calculation process is described in the section of “Methods.” **b** Clusters of differential expressed genes by normalized RPKM accounting the total mRNA content in each sample during pre-implantation development. The first cluster includes 5573 maternal genes, such as ZP family genes, TET3, and GDF9. These 7427 genes from the 2–5 clusters which are activated from the 4-cell stage during the subsequent developmental stages are defined as zygotic genes. The representative GO terms of each cluster and corresponding *P* value are shown at the *right panel*

Next, we estimated the total transcript number and their dynamic changes by using external spike-ins. Considering that the commonly used ERCC spike-ins have low capture efficiency (Additional file 2: Figure S2A), which may be due to their shorter polyA tails (≈20 nt) [20, 21], we added an additional set of RGC-A80 spike-ins for normalization which have 80 nt polyA tails closed to the polyA tail length of the endogenous mRNA molecules (see “Methods”). The accuracy of this algorithm of mRNA quantification was validated by droplet digital polymerase chain reaction (ddPCR) [22, 23] (Additional file 2: Figure S2B). We estimated that a human oocyte expressed an average of 66 million copies of mRNA, which decreased to 29 million after fertilization (Fig. 2a, Additional file 2: Figure S2C–E and Table 1). The lowest total copy number of the mRNAs in each embryo was achieved at the 8-cell stage (average 15.7 million). After this stage, the mRNAs gradually increased in number due to global zygotic activation [24]. In the hatched blastocyst stage, each embryo contained 150 million copies of mRNAs.

**Table 1:** mRNA and circRNA quantification by ERCC/RGC-A80 normalization algorithm

Stage	Oocyte	Zygote	2-cell	4-cell	8-cell	Morula	Early blastocyst	Blastocyst	Hatched blastocyst
Average mRNA copy number (10^6^)	66.4	29.0	24.6	17.0	15.7	38.1	45.9	104.5	150.7
Average circRNA copy number	56,059	48,983	80,410	190,008	148,656	149,508	93,745	40,194	28,774
circRNA/hosting gene (median)	9 %	7 %	9 %	12 %	14 %	25 %	22 %	17 %	20 %
No. of circRNA genes per embryo	149	299	358	727	602	376	519	252	80
No. of circRNA transcript types per embryo	179	392	488	1,115	869	509	630	278	85
No. of circRNA reads per embryo	585	1529	2248	8059	8024	2702	2911	838	225

After normalizing the total mRNA content in each sample during human pre-implantation development, we identified the differential expressed genes between each two stages (upregulated genes: fold-change >2, false positive rate (FDR) <0.05; downregulated genes: fold-change <0.5, FDR <0.05, Additional file 2: Figure S2F). With normalized RPKM, we identified a total of 5573 maternally expressed genes whose expression levels are highest in oocytes and decrease sharply after the 4-cell stage, and a total of 7427 zygotically activated genes whose expression levels elevate prominently during the major wave of MZT (maternal zygotic transition) after the 4-cell stage (Fig. 2b) [1, 24–26]. The maternally expressed genes include the ZP (zona pellucida) family genes and the zygotically activated genes include POU5f1 and TETs genes [1, 25, 27] (Additional file 3: Table S2). Because the embryos from the 4-cell stage and later were developed from cryopreserved embryos, while the embryos of earlier stages were developed from freshly isolated oocytes, it is possible that some changes of the gene expression resulted from the cryopreservation treatment instead.

### Analysis of the circRNAs in human pre-implantation embryos by SUPeR-seq

**Fig. 3:**
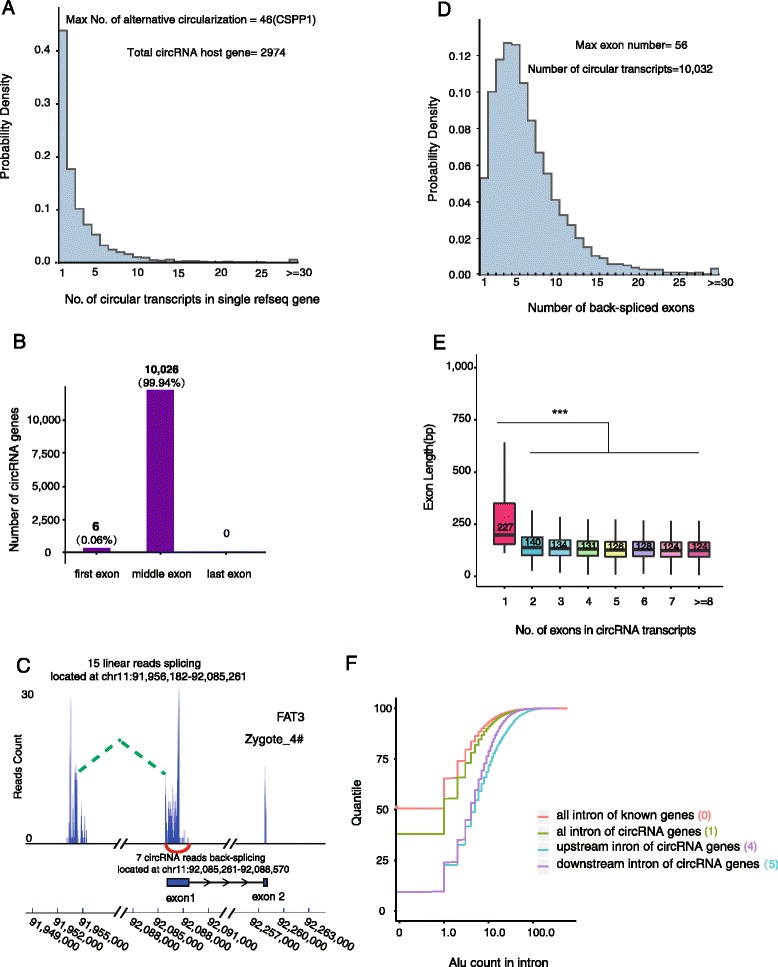
Genomic features of circRNAs expressed during human pre-implantation development. **a** Distribution of the number of different types of circRNA transcripts from each circRNA hosting gene. **b** Distribution of the back-spliced exons in circRNAs. Nearly all (99.9 %) back-spliced exons that contribute to circRNAs are located in the middle of their hosting genes, whereas six are in the first exon and none are in the last exon, as annotated. **c** An example in which potential extra exons are located upstream of the annotated first exon that participates in the circularization of FAT3. Back-spliced reads of FAT3 circRNA are presented as a *red curve*. The peaks connected by the *green dashed line* are the reads mapped to the first exon that participates in the circularization, and the extra exon which is not annotated, simultaneously. **d** Distribution of the number of back-spliced exons in each circRNA. More than 95 % of circRNAs contain multiple back-spliced exons and more than half of them contain 2–6 exons. The maximum number of exons in a single circRNA was 56. **e** Length distribution of back-spliced exons. The *box plots* show that the exon length distribution from the circRNA consisted of a different number of back-spliced exons (***P value = 7.4E-32, Student’s *t*-test). **f** Distribution of the number of Alu elements in flanking and all other introns. The median number is given in the bracket. The number of Alu elements in flanking intron (upstream in *blue* and downstream in *purple*) is much higher than that in the randomly selected control introns no matter in circRNA (in *green*) or not (in *red*)

The circRNA is a new class of polyA– RNAs that has potentially important functions in a variety of biological processes. Using CIRCexplorer, a recently developed software [12], we extracted back-spliced ordering reads from the reads unmapped to the hg19 reference genome. These candidate back-spliced junction reads were then used to annotate exonic circRNAs with precise splice sites linked downstream of the donor exon and upstream of the acceptor exon with at least two back-spliced reads in an individual embryo (see “Methods”). We identified a total of 10,032 exonic circRNAs derived from 2974 hosting genes in human pre-implantation embryos. To validate the strategy for identifying the circRNA, we verified the back-spliced sites from five circRNAs identified in human embryonic stem cells (hESCs) by Sanger sequencing (Additional file 2: Figure S3A and Additional file 4: Table S3). These five circRNA candidates were also resistant to RNase R treatment, which validated their circularized characteristics (Additional file 2: Figure S3B and Additional file 4: Table S3). The abundance of circRNAs dynamically changes between 28,774 and 190,008 copies per embryo during human pre-implantation development (see “Methods” and Table 1). More than half (56 %) of the hosting genes produce multiple circRNA isoforms as “hot-spot” genes (Fig. 3a) [14] and the average expression level for each type of circRNA in each embryo is 92 copies. CSPP1 (centrosome and spindle pole associated protein 1), which has been reported to host a high expression level of circRNAs in porcine embryonic brain tissues [14], produces the highest number of different types of circRNA transcripts during human pre-implantation development (*n* = 46). Previous studies have shown that circRNAs are usually excluded from the first and last exons of their hosting genes [12] and our results showed that this is also true in human pre-implantation embryos: 10,026 (99.9 %) of 10,032 circRNAs have no association with the first or last exons of their hosting genes (Fig. 3b). We then manually examined the six (0.1 %) exceptional circRNAs that appeared to include the first exon and determined that at least five of them had several reads that mapped upstream of the first exon, implying that those originally annotated first exons may not be real first exons. For example, FAT3, which has also been identified as a circRNA hosting gene in another cell line [28] that modulates the extracellular space surrounding axons during embryonic development, seems to produce circRNA containing its first exon. Nevertheless, we detected tens of reads spanning the region upstream of the annotated first exon boundary of FAT3 (Fig. 3c). This confirms the importance of flanking introns for circularization [12, 29, 30]. The majority of circRNAs are composed of multiple exons and the maximum number of exons in a circRNA is 56 (Fig. 3d).

The median length of exons of circRNAs is in the range of 124–227 bp and the longest exons are present in single-exon circRNAs. This observation indicates that a minimum length, approximately 200 bp of hosting RNA, is needed to form a circRNA [12] (Fig. 3e). We observed that the length of introns flanking the circRNAs is prominently longer than control introns (upstream flanking intron: median 8.7 kb; downstream flanking intron: median 7.5 kb, all introns: median 1.6 kb, which is consistent with previous studies [10, 12, 31] (Additional file 2: Figure S3C). While the density of the Alu repeat elements in the flanking introns of circRNAs is similar to control introns (Additional file 2: Figure S3D), the number of Alu elements in the flanking introns is significantly higher than that in control introns (median 4 and 5 versus 1, Fig. 3f), which is consistent with previous findings that Alu element probably promote exon circularization via RNA pairing across flanking introns [12, 29, 32].

### Expression patterns of circRNAs during human pre-implantation development

**Fig. 4:**
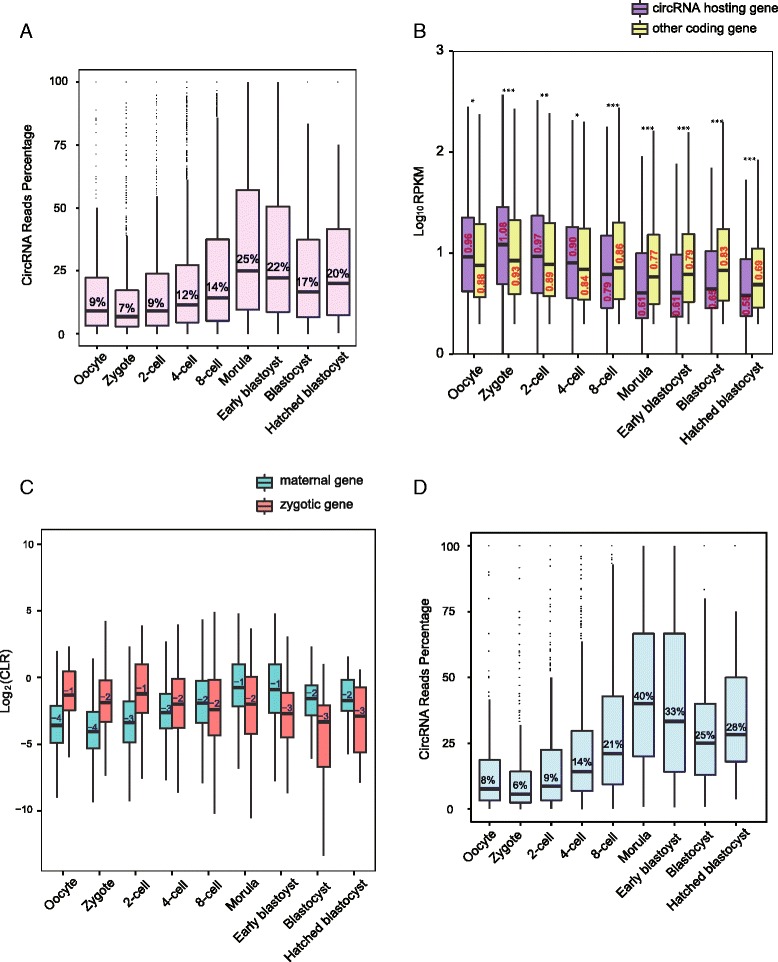
CircRNA and mRNA expression pattern during human pre-implantation development. **a** The pattern of circRNAs percentage in their hosting genes changes during pre-implantation development based on the ratio of back-spliced reads in total reads at each junction locus, the equation is: back-spliced reads/(back-spliced reads + forward-spliced reads). **b** Comparison of the expression levels of circRNA hosting genes and other coding genes in each stage during pre-implantation development. The expression levels of the hosting genes only include linear transcripts by excluding circular transcripts (RPKM_discounted_ = RPKM×(1-circRNA ratio in Fig. 4a). The expression levels of circRNA hosting genes are higher than those of other coding genes before the 8-cell stage, whereas the opposite pattern is observed after the 8-cell stage (***P value <0.001, *P value <0.1, Student’s *t*-test). **c** The CLR values comparison of hosting genes from maternal genes and zygotic genes, respectively, during human pre-implantation development. **d** The circRNA percentage in their hosting genes which are maternal genes and have circular transcripts detected before 4-cell stage, as 950 genes from 1554 maternal hosting genes

Next, we evaluated the ratio of circular transcripts to all transcripts of a given hosting gene by calculating the ratio of back-spliced reads to the total reads mapping to each junction site. In human mature oocytes, circRNAs accounted for on average about 9 % of all transcripts from a hosting gene. This ratio is relatively stable before the 4-cell stage. From the 8-cell stage, the proportion of circRNAs gradually increases, reaching 25 % at the morula stage (Fig. 4a). Thus, circRNAs constitute a significant proportion of hosting gene expression. While the circRNA/hosting gene transcripts ratio is on average approximately 10 %, some circRNAs are expressed at levels even higher than their linear counterparts during pre-implantation development. Five representative genes (PRDM2, SETD2 [5], MLLT3, MLLT4, KIT) are shown in Additional file 2: Figure S4A. These genes participate in different important processes, such as histone methylation and transcriptional regulation.

We also compared the expression levels of circRNA hosting genes with other genes which do not have detectable circular transcripts. Before the 8-cell stage, the averaged expression levels of circRNA hosting genes are significantly higher than those genes that do not have detectable circular transcripts. However, after 8-cell stage, the pattern is reversed (Fig. 4b). In addition, according to their expression patterns during pre-implantation development (Fig. 2b), 2974 circRNA hosting genes can be divided into three clusters: 1554 are maternal genes; 851 are zygotic genes; the remaining 569 genes are undetermined due to our stringent cutoff for the first two clusters. We introduced a parameter, circular to linear ratio (CLR) [13], to compare the relative abundance of a given circRNA to its linear transcripts. During pre-implantation development, the CLR value of the circRNA hosting genes from the maternal gene cluster increases gradually, especially after the 8-cell stage. On the contrary, the CLR value of the circRNA hosting genes from the zygotic gene cluster decreases gradually (Fig. 4c). We also calculated the percentage of circRNA transcripts for the 950 maternal hosting genes who have detectable circular transcripts before the 4-cell stage (Fig. 4d). Comparing the percentage of circRNA transcripts for all hosting genes (Fig. 4a), the increase is sharper and the percentage after the 8-cell stage is higher. These results reflect that circRNAs are more resistant to the global degradation of maternal RNA compared with the linear transcripts during the MZT process [1, 24]. In addition, we made a comparison between the circRNA relative abundance and their hosting gene expression levels. Irrespective of the type of hosting gene, a negative relationship between the logarithm of the CLR value and the hosting gene expression was observed at all time points during human pre-implantation development (Additional file 2: Figure S4B), consistently with the previous finding in neuronal development [13].

Furthermore, we separated all 2974 circRNA hosting genes during human pre-implantation development to ten clusters according to their expression pattern (Additional file 2: Figure S4C and Additional file 5: Table S4). The circRNAs with high CLR values at early stages were mainly enriched for Gene Ontology (GO) terms such as “chromosome organization” and “transcription.” CircRNAs with specifically high CLRs in morula stage embryos are mainly enriched for GO terms including “cell cycle” and “nuclear division.” The corresponding expression levels of these hosting genes are shown in Additional file 2: Figure S4D, with the same clustering manner. In summary, these results showed that more than half of the circRNA hosting genes in human pre-implantation embryos are maternal genes (Fig. 4b, c) and these circRNAs are more resistant to the maternal linear RNA decay machineries than the corresponding linear transcripts (Fig. 4a, 4d and Additional file 2: Figures S4C, D).

### Comparative analysis of human and mouse circRNAs

**Fig. 5:**
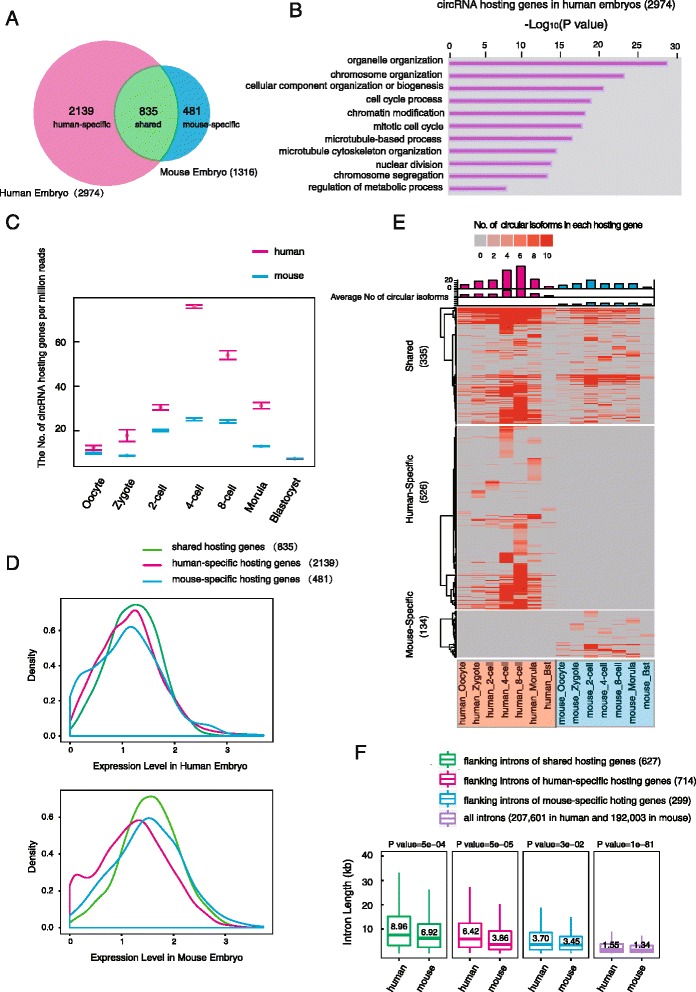
Comparative analysis of human and mouse circRNAs. **a** High conservation of circRNA hosting genes between human and mouse pre-implantation embryos. The *Venn diagram* shows that the majority of genes that express circRNAs in mouse also produce circular transcripts in human embryos. **b** GO analysis of the top enriched terms of the circRNA hosting genes in human pre-implantation embryos. **c** The *box plots* show the numbers of expressed circRNA hosting genes in human embryos (in *red*) and mouse embryos (in *blue*), during pre-implantation development, and the number was normalized to the total mapped reads (in millions) after subsampling the sequencing data. **d** Expression level of circRNA hosting genes in human embryo and mouse embryo. All circRNA hosting genes are plotted according to the value of log_10_ of (max RPKM during pre-implantation) in human and mouse embryo, respectively. **e** *Heat map* of circularized level of highly expressed circRNA hosting genes (RPKM >10) during human and mouse pre-implantation development. The numbers of circular isoforms from each hosting gene were presented and the *columns* on the top of the *heat map* show the average number of circular isoforms from each hosting gene who were producing circular transcripts at this stage. The number of circRNA isoforms is frequently higher than that in mouse. The shared, human-specific, and mouse-specific hosting genes are presented, respectively. **f** *Box plots* show the distribution of the length of introns flanking the back-spliced sites from the highly expressed hosting genes after subsampling the sequencing data. The median value of intron length is shown in the box. The back-spliced sites of human-specific and mouse-specific circRNAs are converted using liftover tool, respectively. The numbers of introns are indicated in the bracket

To gain insight into the evolution of circRNAs, we compared the circRNAs of human pre-implantation embryos with those identified in our previous mouse study [19]. Of all the 1316 circRNAs hosting genes identified in mouse pre-implantation embryos, 835 (63 % of 1316) also generate circRNAs in human embryos, indicating that the circRNA production is in general conserved between human and mouse (Fig. 5a). Of the 2926 circRNA hosting genes identified in human H9 embryonic stem cells (ESCs) in a previous study using a different method (Additional file 6: Table S5) [12], 1388 (47 % of 2926) were found to generate circRNAs in human early embryos (Additional file 2: Figure S5A). GO analysis against the background linear RNA expression showed that the circRNAs hosting genes in human embryos are enriched for "genes of organelle organization", "chromosome organization", "cell cycle process", and "regulation of metabolic process", which is similar to those in mouse embryos and H9 cells (Fig. 5b, and Additional file 2: Figure S5B, C). These results indicate that circRNAs are generated by a highly conserved set of genes in both human and mouse.

An interesting finding is that human-specific circRNAs hosting genes prominently outnumber the mouse-specific ones (human versus mouse: 2139 versus 481, Fig. 5a, Additional file 2: Figure S5D). To exclude the effect of different sequencing depth, we subsampled the human and mouse sequencing data to the same depth and obtained a similar result (human versus mouse: 795 versus 285, Additional file 2: Figure S5E). The human early embryos showed a higher number of circRNA hosting genes as well as more types of circRNA transcripts than the mouse embryos from oocyte to morula stages, reaching the highest level at the 4-cell stage (Fig. 5c and Additional file 2: Figure S5F). Comparing expressions of the species-specific and shared genes showed that, while species-shared circRNAs hosting genes are generally expressed in both human and mouse embryos, a portion of species-specific circRNA hosting genes are solely expressed in the corresponding species (Fig. 5d). This indicates that the species-specific circRNAs are partially due to the differential expression of their hosting genes between human and mouse pre-implantation embryos.

To investigate whether there were factors other than differential hosting gene expression leads to a species difference of circRNA, we compared the circRNAs derived from the hosting genes that are highly expressed in both human and mouse embryos (RPKM >10, Additional file 2: Figure S5G). The result showed that the human-specific circRNA hosting genes still outnumber the mouse-specific ones (human versus mouse: 526 versus 134, Fig. 5e) and also in the subsampling data (human versus mouse: 232 versus 104, Additional file 2: Figure S5H). Since the flanking intron has been shown to play an important role in circRNA generation, we calculated the length of introns flanking these circRNAs. Notably, we found that the introns flanking the species-shared or human-specific circRNAs are significantly longer than their mouse counterparts (*P* <0.001, Fig. 5f). In particular, the introns flanking the human-specific circRNAs in the human genome are about 1.7-fold longer than their mouse counterparts (human versus mouse: median 6.42 versus 3.86 kb, *P* = 5E-5, Fig. 5f). On the contrary, the length of the introns flanking the mouse-specific circRNAs showed only a mild difference between the two species (human versus mouse: median 3.7 versus 3.45 kb, *P* = 0.03).

Together, these results demonstrated that circRNAs in human pre-implantation embryos are more complex compared with those in mouse embryos, which may be partially due to the increase in intron length during evolution of the human genome.

### Analysis of the novel linear transcripts in human pre-implantation embryos by SUPeR-seq

**Fig. 6:**
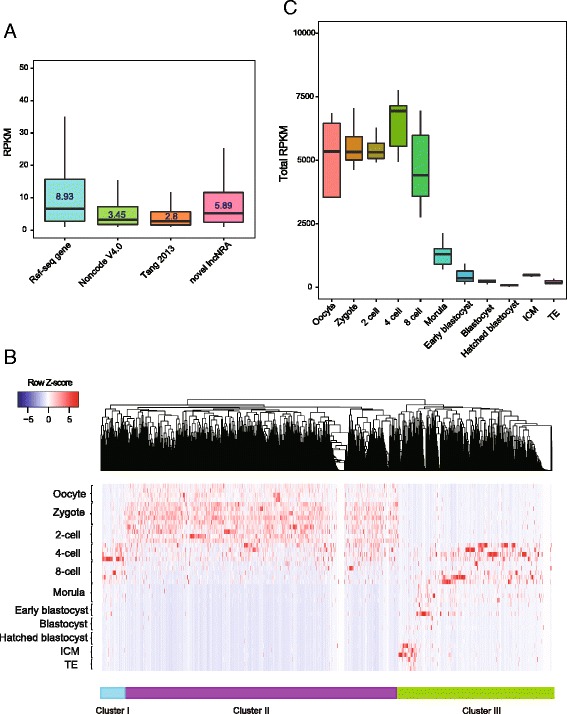
Expression pattern of *de novo* assembled transcripts during human pre-implantation development. **a** The *box plots* show that the expression levels of the novel assembled genes are lower than those of the RefSeq genes but higher than those of the lncRNAs from Noncode V4.0 and our previous paper. **b** Hierarchical clustering analysis of novel genes indicating stage-specific expression patterns during human pre-implantation. Based on the heat map, these novel genes can be divided into three major types: early zygotic genes, maternal genes, and late zygotic genes. **c** The pattern of total expression levels of all novel 2322 candidate genes revealing that genes enriched before the 8-cell stage are mostly maternal genes

Finally, we performed RNA *de novo* assembly and identified 2322 novel candidate transcript units and 10,084 candidate isoforms by excluding known genes in RefSeq genes, Noncode V4.0 long non-coding RNA (lncRNA) databases [33], and novel lncRNAs reported in our previous study [1] (Additional file 7: Table S6). These *de novo* transcripts were rigorously filtered: each transcript comprised at least two exons, was located >10 kb away from the known genes in the human genome, and was longer than 500 bp [1, 19, 34]. The average length of these candidates was 1052 bp and they were usually shorter than 2 kb (Additional file 2: Figure S6A). These novel genes have longer introns than other known genes and half of them consist of two exons (Additional file 2: Figures S6B and S6C). Furthermore, their average expression levels are higher than those of the genes identified in the Noncode database or our previous work using the single-cell RNA-seq technique that only detected polyA+ mRNAs (Fig. 6a), implying that these novel transcripts are polyA– or with short polyA tail. These novel genes were classified into distinct categories that may function in specific stages during pre-implantation development (Fig. 6b). The expression levels of novel transcripts in each developmental stage increased before the 8-cell stage and then decreased (Fig. 6c). This pattern was similar to that of the novel genes discovered in mouse embryos using SUPeR-seq [19]. The conservation level (calculated with the metric ω) [35] of these novel transcripts was similar to those of the known lncRNAs and novel transcripts that we detected previously. In addition, these novel transcript candidates were less conserved than protein-coding exons but more conserved than the introns of protein-coding genes (Additional file 2: Figure S6D). CPC (Coding Potential Calculator) [36] analysis revealed that among these 2322 novel genes, 89.9 % (2087) produced transcripts without significant coding potential, indicating that they were potential novel lncRNAs.

## Conclusions

Overall, our investigation of the transcriptomic landscape of human pre-implantation development by SUPeR-seq identified abundant circRNAs and revealed dynamic gene expression changes during human pre-implantation development. A large number of circRNAs are transcribed from maternal genes, most of which are present before fertilization, and persisted during pre-implantation development possibly due to their resistant to the maternal mRNA degradation process. Compared with mouse, human circRNAs are proved to have both conservation and an increase in complexity, pointing to their conserved and specific roles during human pre-implantation development. In sum, our data provide an invaluable resource for investigating their functions in the future.

## Methods

### Embryo collection

The oocytes and embryos for this study were donated from female volunteers who provided informed consent. After ICSI (intracytoplasmic sperm injection), embryos were cultured in G1.3 medium (Vitrolife, Sweden) covered with mineral oil (Sigma, 6 % CO_2_). Oocytes, zygotes, and 2-cell-stage embryos were collected at the appropriate time during embryonic development.

Embryos at the 4-cell and 8-cell stages were thawed immediately after removal from liquid nitrogen as described previously [1]. The embryos were cultured in G2 medium (Vitrolife) continuously to obtain morulae and early blastocysts, blastocysts, and hatched blastocysts.

Each selected oocyte or embryo was transferred drop wise to the acidic solution to remove the zona pellucida by mouth pipette. Then, the embryo or oocyte was washed gently several times before being transferred to lysis buffer.

To obtain ICM and TE transcriptome information, we isolated these compartments from each other by laser cutting. This process was executed carefully to retain all cells in the ICM with minimal laser damage.

### Single embryo transcriptome amplification

The RNAs in individual oocytes or embryos were reverse transcribed and amplified using the SUPeR-seq method we recently developed. Briefly, after cell lysis, RNAs with or without polyA were reverse transcribed with T15N6 primer [19] using Super Script III (Invitrogen). After reverse transcription, unreacted primer was digested by ExoSAP-IT (USB) and RNA was degraded by RNase H (Invitrogen). Then, a polyA tail was added to the first strand cDNA at its 3′ end by terminal deoxynucleotidyl transferase (Invitrogen). Thus, the second strand cDNA could be synthesized by a primer with a poly T and an anchor sequence. The double-stranded cDNAs were then amplified by primers with the two anchor sequences for 20 + 10 cycles.

Before single-cell RNA amplification, we quantitatively added spike-in RNAs, as ERCC RNA Spike-In Mix1 (Ambion) and RGC-A80 to the lysis mixture. The spike-in RNAs were used for quality control and mRNA quantification.

### RNA-seq library construction and sequencing

After the single-cell cDNAs were amplified with the SUPeR-seq method, we sheared approximately 200 ng of purified cDNA products into fragments of 150–350 bp using the Covaris S2 system. The fragmented DNA was subjected to end-repair, dA-tail, adaptor ligation, and 10–12 cycles of PCR amplification using the TruSeq DNA library preparation kit (Illumina).

### SUPeR-seq data processing and validation of circRNA candidates

The sequenced raw data were first cleaned to remove low-quality reads (reads with more than 50 % of the bases with quality value ≤5 and >10 % of the bases undetermined). The adaptor sequences, poly (A) 24/(T) 24 sequences and sequences with >80 % AT bases were trimmed. To detect circular reads using CIRCexplorer, the trimmed data were aligned to an hg19 reference using the two-step approach recommended by https://github.com/YangLab/CIRCexplorer/ [12].

The mapped reads in the first step were considered linearly mapped reads. For linearly mapped reads, HTSeq [38] was used to count the unique mapped reads to each gene to estimate the abundance of the transcripts (shown as RPKM) and define differentially expressed genes. We used a GTF file combined with hg19 RefSeq genes in the UCSC Genome Browser, the NONCODE V4.0 database, and the genes from a former study previously reported by our lab [1] to identify non-coding genes as well as 92 exogenous ERCC spike-in RNAs and RGC-A80 information. After HTSeq, unannotated reads were used to assemble novel genes. The potential novel transcripts were identified based on three criteria. First, the expression level (RPKM) of candidate transcripts was >0.5 and the RPKM in every replicate was >0.25. Second, the potential novel transcript was at least 10 kb away from any known genes. Third, the potential novel gene had at least two exons, and the total length for all exons was >500 bp. The coding potential for novel genes was estimated using the Coding Potential Calculator (http://cpc.cbi.pku.edu.cn) [36].

In the second step, the unmapped reads from the first step were mapped to the genome using TopHat-Fusion [12, 39]. CIRCexplorer was used to detect circular reads. Because we used pair-end sequencing data, which provides more reliable results for circular regions, CIRCexplorer was modified to ensure that each circular read had a back-spliced read across two exon junctions in the same gene and the other read from a pair-end reads was linearly aligned between the two exons. Finally, the ratio of circular to linear transcripts was estimated by the back-spliced reads over the step1 mapped reads at each junction locus.

We verified five circRNA candidates in hESCs. The total RNA was extracted from 1 million hESCs, then the total RNA (2 ug) was treated with RNase R (Epicentre) or nuclease-free water as mock control at 37 °C for 15 min. After being reverse transcribed with random primers, the cDNAs were used as qPCR templates to compare the different effects of RNase R treatment between the linear transcripts and circRNA candidates. The hESCs total RNA was subjected to RT-PCR and Sanger sequencing to verify the back-spliced sites of circRNA candidates at single-base resolution.

### Estimation of technical error

We first merged the counts of reads mapped to the 92 exogenous ERCC spike-in RNAs for each sample. Then, the ERCC expression level (RPKM) matrix was calculated using the total mapped reads and the length of each spike-in molecule. Only ERCC with RPKM ≥1 in more than two samples was considered. ERCC with RPKM <1 was excluded for further analysis. The technical error was then estimated using the Pearson correlation between samples.

### Correlation analysis for RNA-seq data and hierarchy clustering, PCA

The correlation between samples was calculated using the RefSeq gene expression level (RPKM) matrix with the parameter *use = “pairwise.complete.obs,” method = “pearson”* using an in house-developed R script. Based on the correlation matrix, ward distance was used when performing hierarchy clustering. PCA was also performed using the FactoMineR package in the CRAN R program based on the same expression level matrix.

### Quantification of total transcripts copy number

The spike-ins of RGC-A80 included three species of in vitro transcribed RNA molecules (RFP, GFP, and CRE) which had 80 nt polyA tails and mixed as the molecule ratio of RFP: GFP: CRE being 100: 10: 1. By using ddPCR (BioRad, QX200), the capture efficiency of RGC-A80 was verified as about three times higher than ERCCs in SUPeR-seq (Additional file 2: Figure S2A). Therefore, addition of the RGC-A80 spike-ins can partially overcome the low capture efficiency of the ERCC spike-ins and achieve a more accurate estimation of the total transcript content in each sample.

For the ERCC/RGC-A80 normalization algorithm, firstly, linear regression was applied to fit the data points between the RPKM value of the 92 exogenous ERCC spike-in RNAs (log10-transformed RPKM) in the SUPeR-seq dataset and the number of molecules per lysis reaction (log10-transformed attomole) (Additional file 2: Figure S2C). Only ERCC species whose molecules >0.001 attomole were retained in the regression. The linear regression equations for each sample were then applied to the RPKM value of all RefSeq genes and summed up as the ERCC-based total mRNA copy number. Then, the total mRNA copy number was also calculated from each molecule species from spiked RGC-A80 as the ratio of the total RPKM to the RGC-A80 RPKM multiply the spiked molecules and then averaged out (Additional file 2: Figure S2D). Lastly, the final total mRNA copy number was obtained by fitting the RGC-A80-based and the ERCC-based values in all 29 samples to a linear regression model (Additional file 2: Figure S2E).

We also estimated the copy number of circRNAs in each oocyte and embryo during human pre-implantation development. Firstly, we calculated the RPKM of circRNA as junction reads/(circRNA length × total mapped reads). And the circRNA length = (length of reads-25 bp) × 2, as 25 bp is the segment length of Tophat. This means that for reads of 100 bp in length, a back-splicing event can be detected by reads mapping up to 75 bp away in each direction, as 75 bp × 2 = 150 bp in length. Then we could calculate the copy number of circRNAs = (sum of circRNAs’ RPKM)/(sum of Refseq genes’ RPKM) × (copy number of mRNA).

### Copy number quantification with ddPCR

For validation of the algorithm of mRNA quantification in SUPeR-seq by ERCC and RGC-A80, the total RNAs were extracted from ~100,000 hESCs and SUPeR-seq was performed in three technical replicates for 1 ng total RNA in each replicate. The rest of the RNAs were reverse transcribed to cDNAs to examine the copy number of 44 genes in 1 ng RNAs by ddPCR in two replicates. The primers for ddPCR are listed in Additional file 8: Table S7.

### Differentially expressed genes identified based on normalized RPKM

Differential gene expression analysis across all samples was performed using the DESeq2 package in the Bioconductor R program [40], which is based on the negative binomial distribution model. Raw read counts calculated by HTSeq were normalized by a set of size factors accounting for both the sequencing depth and the mRNA quantity in each sample. For a strict definition of differential expressed genes, the RefSeq genes expressed in at least one of the samples with normalized RPKM ≥1 were used for the analysis.

### Comparison of circRNAs with hESC data and mouse pre-implantation embryo data

The hosting gene and read counts of the circRNA matrix were merged from the pair-end checked CIRCexplorer results. For each sample, circRNAs with read depths <2 were discarded. The hosting gene of circRNAs in H9 cell line were filtered according to the gene list provided in a former study [12] and are listed in the Supplemental Information Table. We also subjected the sequencing data of mouse early embryo cell data in our published work [19] to the same approach. The 20 bp at each site of the back-spliced junction of mouse circRNAs were converted from mm10 to hg19 using the *liftover* tool from UCSC utilities with the parameter *-minMatch = 0.5*, which enabled comparison with human early embryo cell data [14]. When comparing the circRNA expression pattern, we subsampled the sequencing data from human and mouse pre-implantation embryos to the same depth as 9 millions reads per stage and kept the same number of developmental stages side by side for analysis. Venn diagrams were used to show the shared gene lists and GO analysis for each portion of the Venn diagram was performed using the GOstat package in the Bioconductor R program [41]. For GO analysis of the circRNA hosting genes in human pre-implantation embryos, all genes expressed above 1 RPKM were set as the background. The combined gene list of circRNA hosting genes in human and mouse embryos was set as the background for the comparison between these two species. The combined gene list of circRNA hosting genes in human embryos and H9 cells was set as the background for the comparison between these two cell sources.

## Abbreviations

circRNA, Circular RNA; CLR, Circular to linear reads ratio; ddPCR, Droplet digital polymerase chain reaction; DEG, Differential expressed genes; ERCC, External RNA Controls Consortium; FDR, False positive rate; GO, Gene ontology; hESCs, Human embryonic stem cells; lncRNA, long non-coding RNA; MZT, Maternal to zygotic transition; PCA, Principal component analysis; RGC-A80, Mixture of RFP, GFP, CRE with 80 nt polyA tails; RPKM, Reads per kilobase of exon model per million mapped reads; RT, Reverse transcription; SUPeR-seq, Single cell universal poly(A)-independent RNA sequencing

## Additional files

Additional file 1: Table S1. Sequencing statistics summary of samples analyzed in this study. (XLS 65 kb) Additional file 2:
**Figure S1.** Reproducibility of the SUPeR-seq method. **Figure S2.** mRNA copy numbers quantified by ERCC/RGC-A80 normalization algorithm. **Figure S3.** Feature analysis of the flanking introns of circRNAs. **Figure S4.** Expression pattern of circRNAs and the hosting genes during human pre-implantation development. **Figure S5.** Comparison of circRNA hosting genes. **Figure S6.** *De novo* assembled transcripts in the human pre-implantation embryos. (PDF 23698 kb) Additional file 3: Table S2. Normalized RPKM of RefSeq gene. Corresponding to Fig. 2b, the 5573 RefSeq genes clustered in group 1 were defined as maternal genes and the 7427 genes in groups 2 to 5 transcribed *de novo* at later stages from the late 4-cell stage were defined as zygotically activated genes. (XLSX 1359 kb) Additional file 4: Table S3. Sequencing statistics and circRNA candidates of hESCs in this study. (XLS 77 kb) Additional file 5: Table S4. CLR value of circRNA during human pre-implantation development. The log2-transformed values of CLR for all circRNA hosting genes at all developmental stages were listed. NAN means this hosting gene have not circRNA reads at this stage. (XLSX 245 kb) Additional file 6: Table S5. Circular RNA detected in human embryos and list of circRNA hosting genes in H9 Cell. The circRNA hosting genes in H9 cell line were filtered as the reads counts >1 according to the Supplemental Table in a previously published paper [12]. (XLS 1349 kb) Additional file 7: Table S6. A total of 2322 novel assembled gene RPKM cluster and transcripts locus information. (XLS 4066 kb) Additional file 8: Table S7. Sequences of RGC-A80 and the primers used in ddPCR and circRNAs validation. The sequences of RGC-A80, the primers used in ddPCR for detecting the copy number of 44 genes, and the primers of circRNAs validation are listed. (DOCX 19 kb)
