# Supplementary material for: Tracing the expression of circular RNAs in human pre-implantation embryos
Source: Genome Biol. 2016 Jun 17;17:130. doi: 10.1186/s13059-016-0991-3 (PMC4911693; doi:10.1186/s13059-016-0991-3)
Supplement: Additional file 2: — Figure S1. Reproducibility of the SUPeR-seq method. Figure S2. mRNA copy numbers quantified by ERCC/RGC-A80 normalization algorithm. Figure S3. Feature analysis of the flanking introns of circRNAs. Figure S4. Expression pattern of circRNAs and the hosting genes during human pre-implantation development. Figure S5. Comparison of circRNA hosting genes. Figure S6. De novo assembled transcripts in the human pre-implantation embryos. (PDF 23698 kb) [file 13059_2016_991_MOESM2_ESM.pdf]

Supplementary Information

A

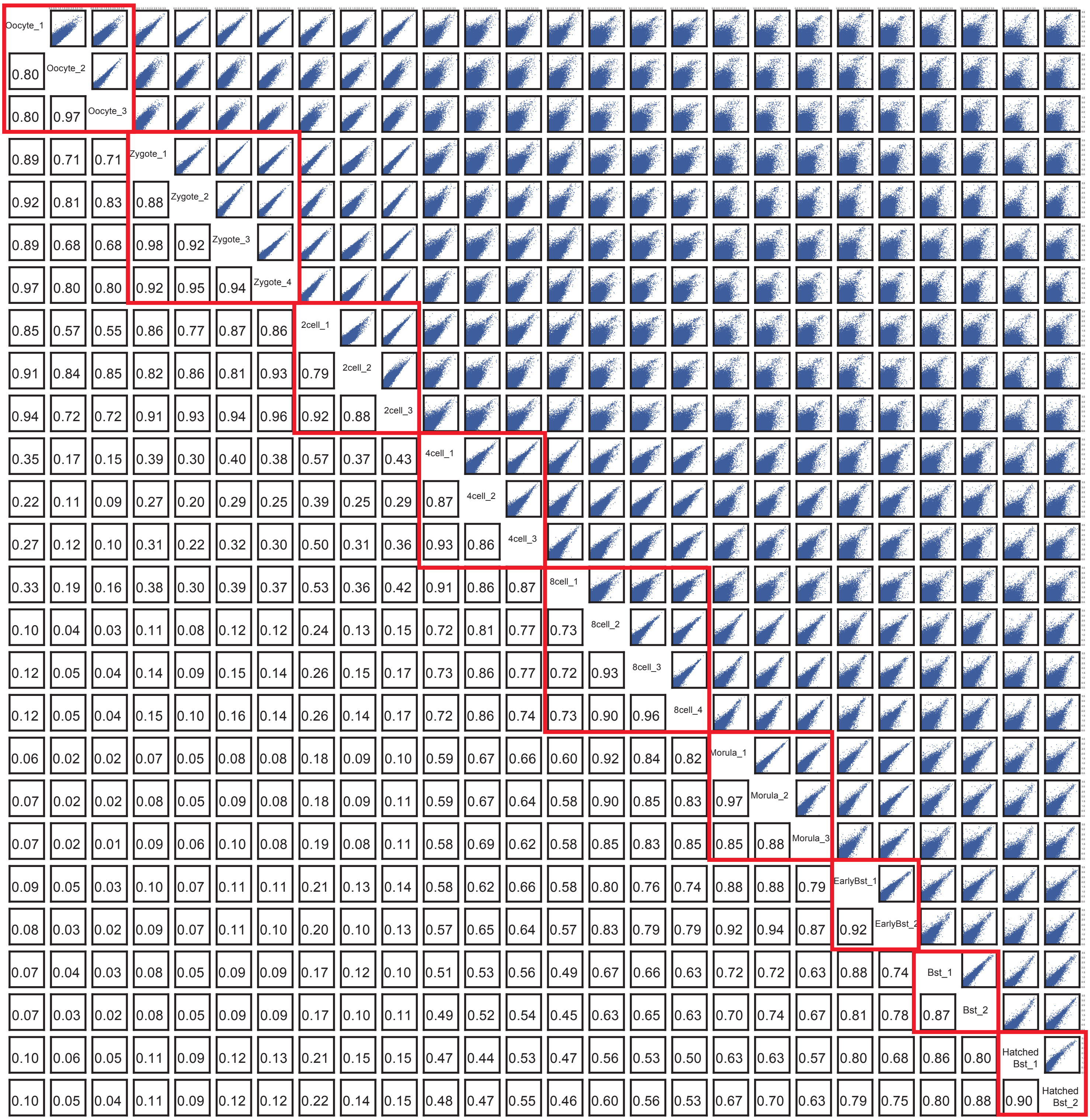

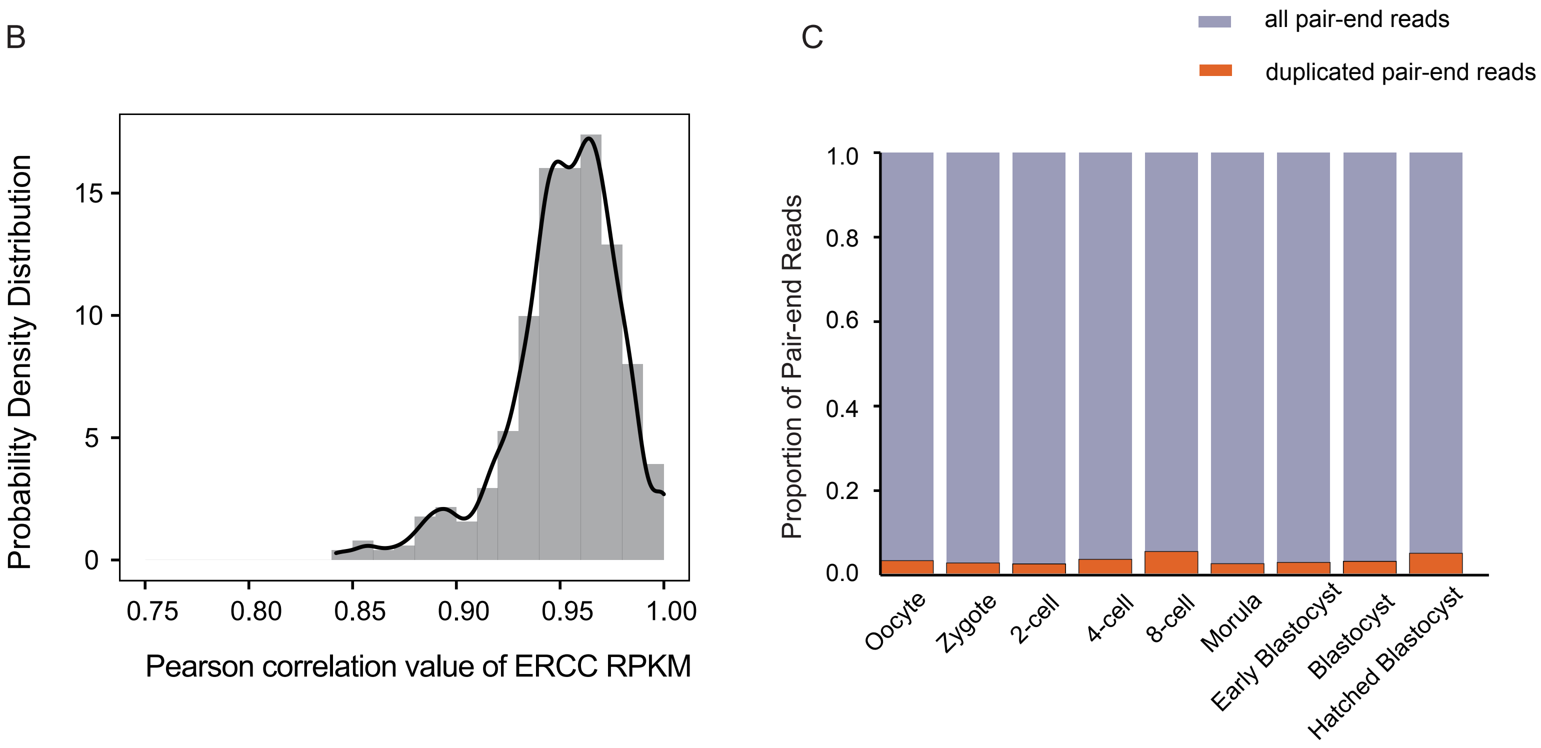

Figure S1. Reproducibility of the SUPeR-seq method.

(A) Pearson correlation coefficients of oocytes and embryos during human pre-implantation development. R squared value of pair-wise correlation between each two samples are shown. Blastocyst is abbreviated to Bst.

(B) Distribution of Pearson correlation coefficients of ERCC spike-in RNAs during human pre-implantation development.

(C) Proportion of duplicated pair-end reads in all pair-end reads by SUPeR-seq of human early embryos.

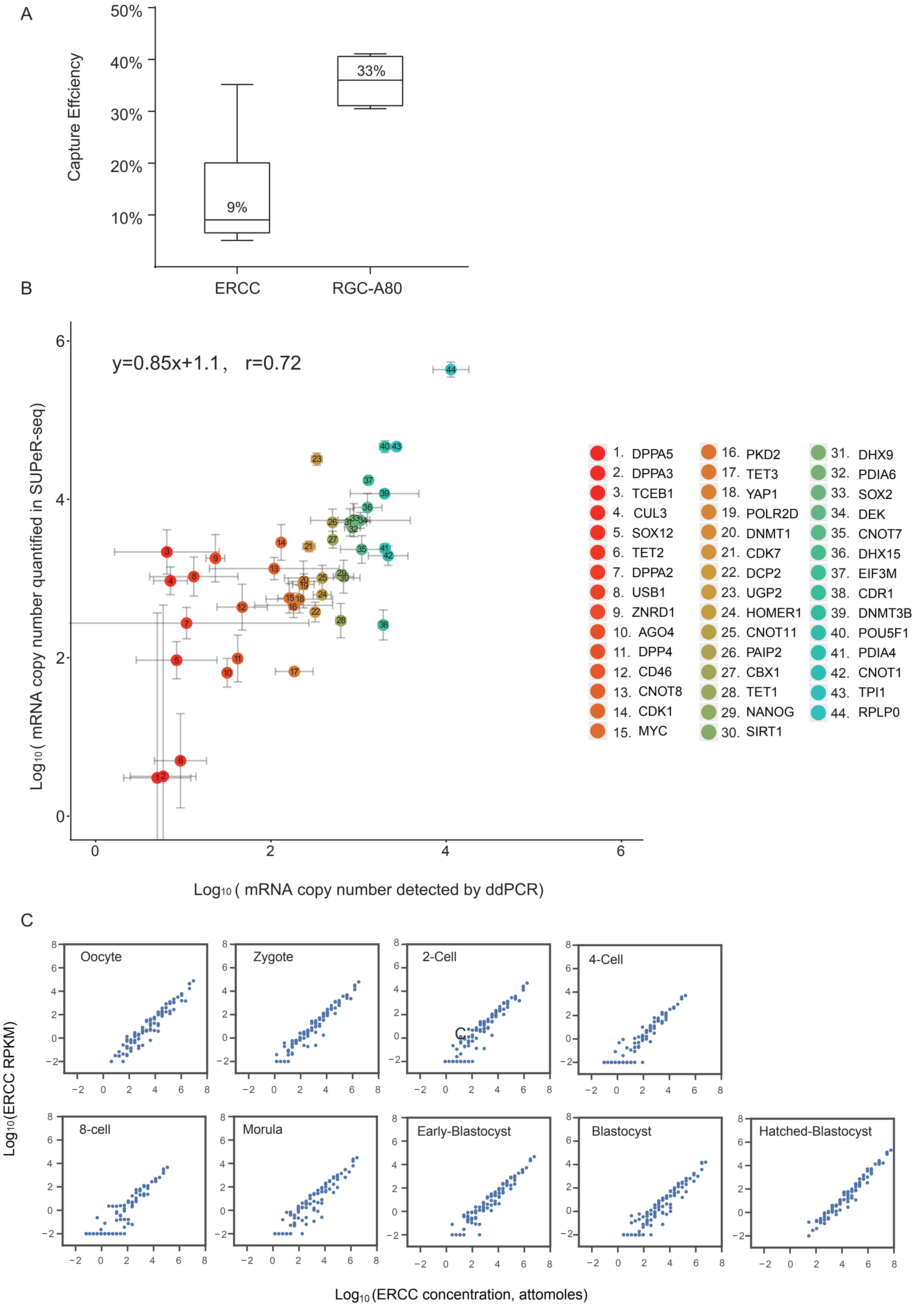

A
Capture Effciency
50%
40%
30%
20%
10%
33%
9%
ERCC
RGC-A80
B
y=0.85x+1.1， r=0.72
Log10 (mRNA copy number quantified in SUPeR-seq)
Log10 (mRNA copy number detected by ddPCR)
1. DPPA5
2. DPPA3
3. TCEB1
4. CUL3
5. SOX12
6. TET2
7. DPPA2
8. USB1
9. ZNRD1
10. AGO4
11. DPP4
12. CD46
13. CNOT8
14. CDK1
15. MYC
16. PKD2
17. TET3
18. YAP1
19. POLR2D
20. DNMT1
21. CDK7
22. DCP2
23. UGP2
24. HOMER1
25. CNOT11
26. PAIP2
27. CBX1
28. TET1
29. NANOG
30. SIRT1
31. DHX9
32. PDIA6
33. SOX2
34. DEK
35. CNOT7
36. DHX15
37. EIF3M
38. CDR1
39. DNMT3B
40. POU5F1
41. PDIA4
42. CNOT1
43. TPI1
44. RPLP0
C
Oocyte
Zygote
2-Cell
4-Cell
8-cell
Morula
Early-Blastocyst
Blastocyst
Hatched-Blastocyst
Log10(ERCC RPKM)
Log10(ERCC concentration, attomoles)

D

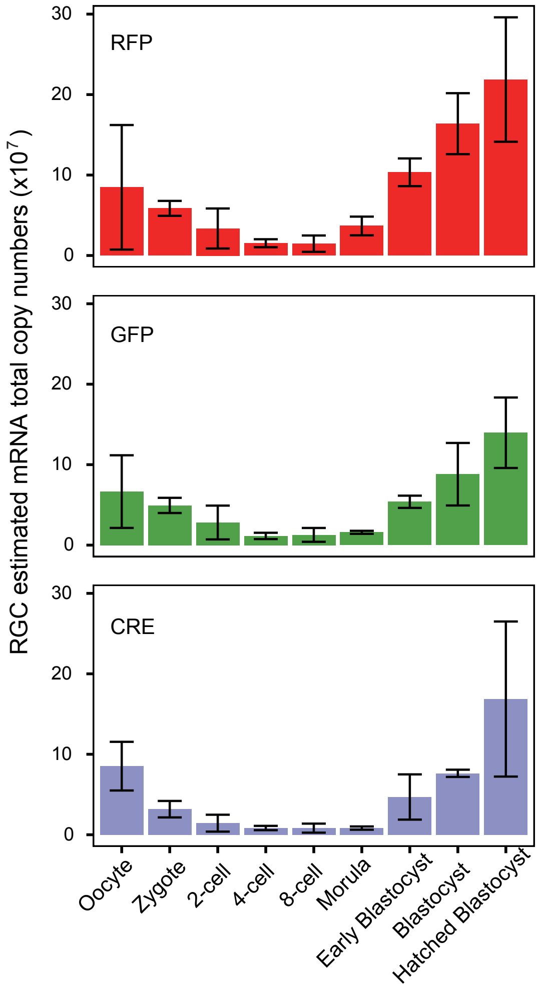
RFP
GFP
CRE
RGC estimated mRNA total copy numbers (x10^7)
0
10
20
30
Oocyte
Zygote
2-cell
4-cell
8-cell
Morula
Early Blastocyst
Blastocyst
Hatched Blastocyst

E

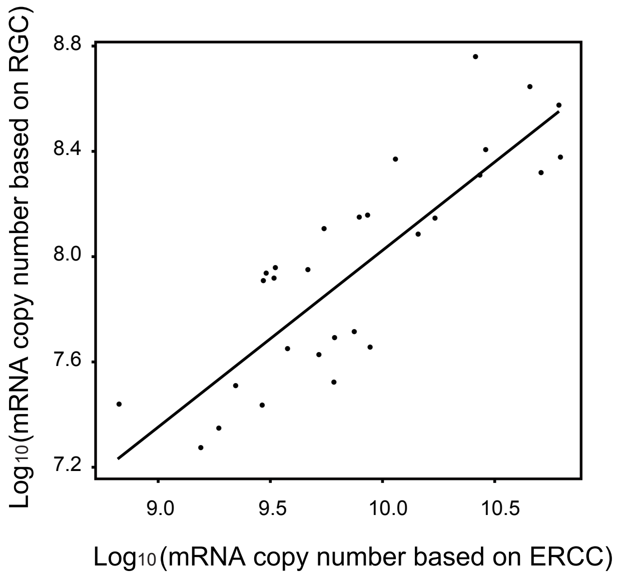
Log10(mRNA copy number based on RGC)
8.8
8.4
8.0
7.6
7.2
9.0
9.5
10.0
10.5
Log10(mRNA copy number based on ERCC)

F

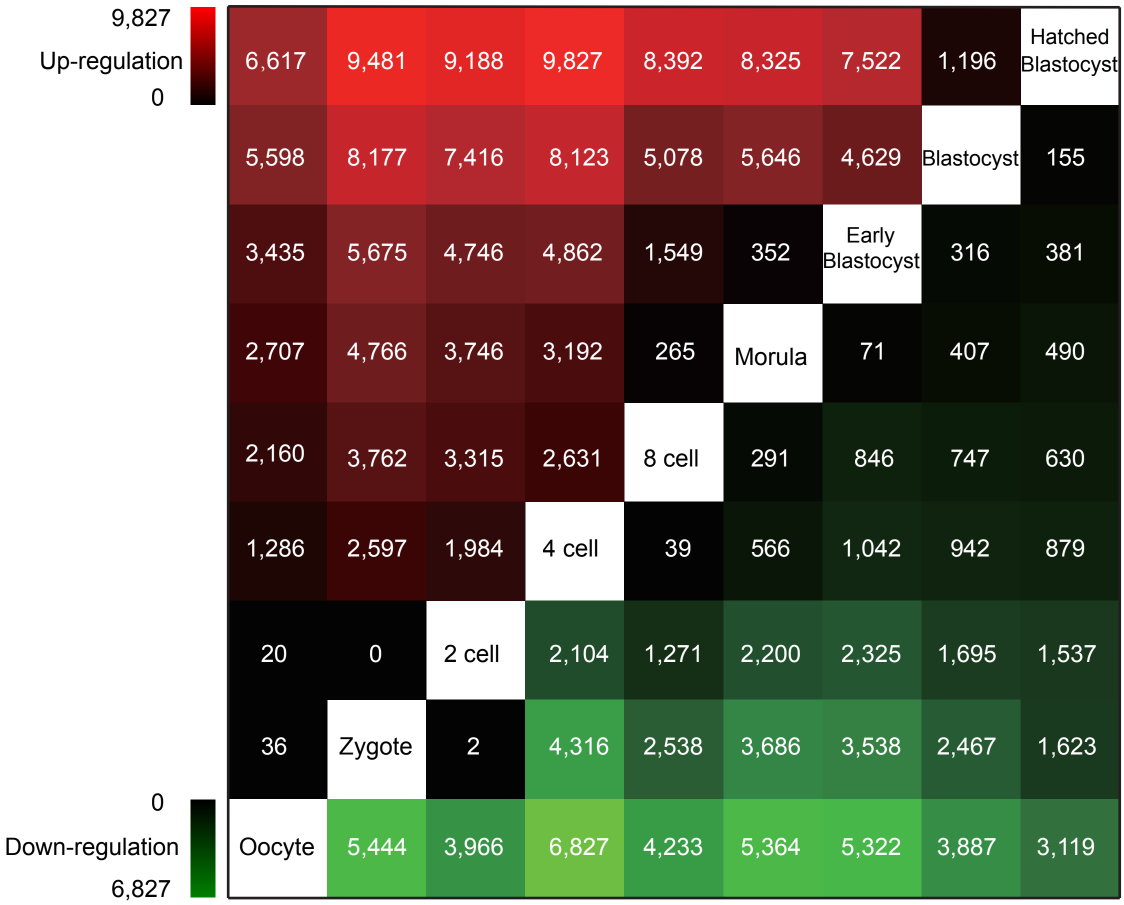
9,827
Up-regulation
0
0
Down-regulation
6,827
6,617 9,481 9,188 9,827 8,392 8,325 7,522 1,196 Hatched Blastocyst
5,598 8,177 7,416 8,123 5,078 5,646 4,629 Blastocyst 155
3,435 5,675 4,746 4,862 1,549 352 Early Blastocyst 316 381
2,707 4,766 3,746 3,192 265 Morula 71 407 490
2,160 3,762 3,315 2,631 8 cell 291 846 747 630
1,286 2,597 1,984 4 cell 39 566 1,042 942 879
20 0 2 cell 2,104 1,271 2,200 2,325 1,695 1,537
36 Zygote 2 4,316 2,538 3,686 3,538 2,467 1,623
Oocyte 5,444 3,966 6,827 4,233 5,364 5,322 3,887 3,119

Figure S2. mRNA copy numbers quantified by ERCC/RGC-A80 normalization algorithm.

(A) Capture efficiency of ERCC and RGC-A80 during reverse transcription in SUPeR-seq tested by ddPCR. These two spike-ins were reverse transcribed at same masses following the protocol of SUPeR-seq, and the unamplified cDNA were used as templates in ddPCR reaction for ten ERCC genes, and RFP and GFP twice independently.
(B) Validation of the quantification in SUPeR-seq through detection the copy number of 44 genes in 1ng hESCs total RNAs by ddPCR. The copy numbers of these 44 genes estimated in SUPeR-seq were distributed from the total mRNA copy number by the ratio of each gene's RPKM to the total RPKM of all RefSeq genes. The correlation between the data from ddPCR and SUPeR-seq is 0.72. The linear regression formula based on these genes is y=0.85x+1.1. Errors bars show the 95%CI for individual ddPCR measurements (horizontal bars, n=2) or across SUPeR-seq (vertical bars, n=3).
(C) Linear regression of 92 ERCCs between the copy number and RPKM at each stage.
(D) mRNA copy number of RefSeq genes per embryo estimated based on RGC-A80 spike-ins.
(E) Linear regression of the mRNA copy numbers per embryo based on two sets of spike-in molecules, r=0.71. The linear regression equation is y=0.67x+1.3, and x represents the log10-transformed ERCC estimated mRNA copy number, and y represents the log10-transformed RGC-A80 estimated mRNA copy number. The final mRNA copy number per embryo was the value for y that was calculated using the linear regression equation for each value of x.
(F) Number of known RefSeq genes showing up-regulation and down-regulation during human pre-implantation development after size-factoring the sequencing depth and the mRNA quantity.

A

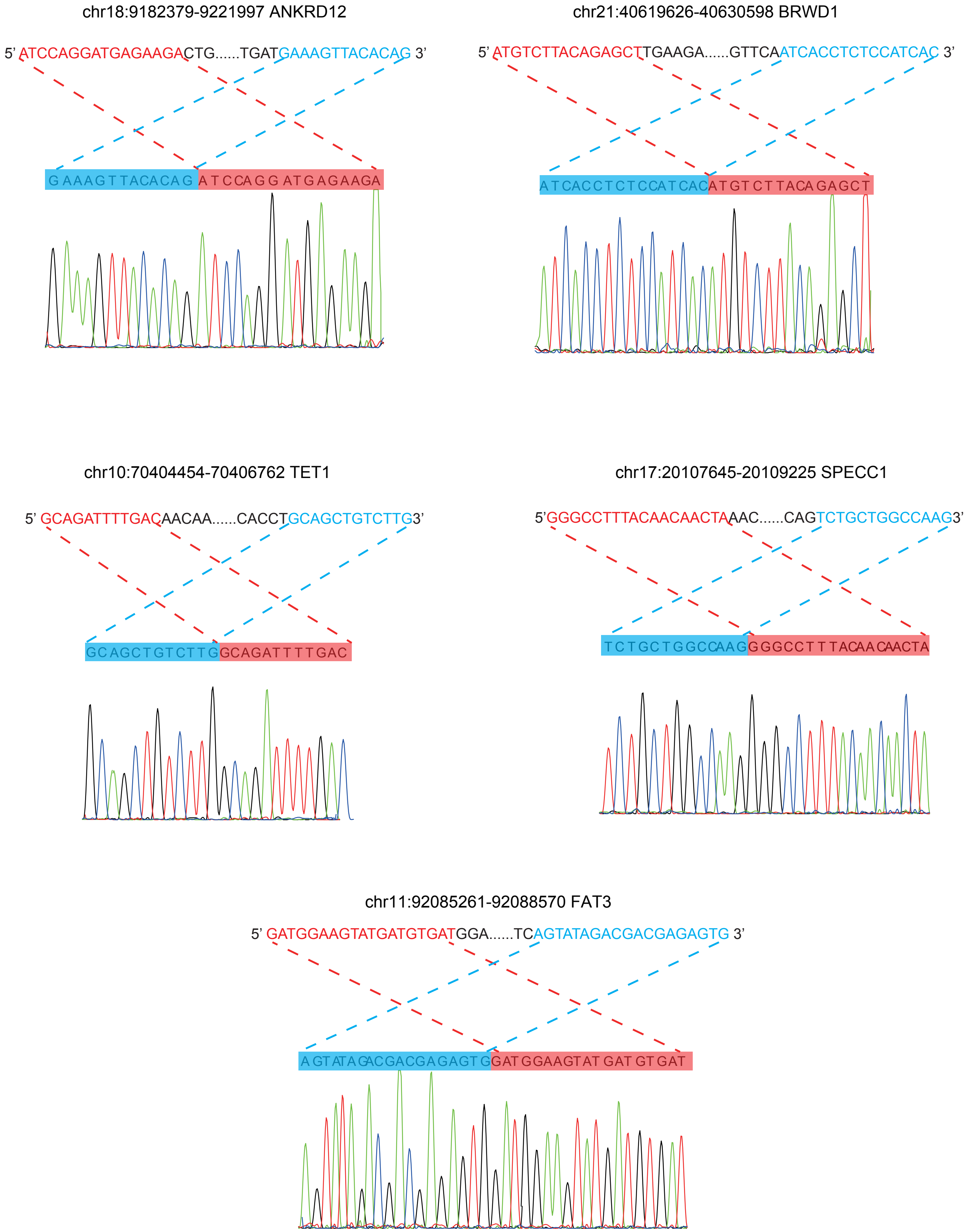

chr18:9182379-9221997 ANKRD12
5' ATCCAGGATGAGAAGACTG......TGATGAAAGTTACACAG 3'
GAAAGTTACACAG ATCCAGGATGAGAAGA
chr21:40619626-40630598 BRWD1
5' ATGTCTTACAGAGCTTGAAGA......GTTCAATCACCTCTCCATCAC 3'
ATCACCTCTCCATCAC ATGTCTTACAGAGCT
chr10:70404454-70406762 TET1
5' GCAGATTTTGACAACAA......CACCTGCAGCTGTCTTG3'
GCAGCTGTCTTG GCAGATTTTGAC
chr17:20107645-20109225 SPECC1
5'GGGCCTTTACAACAACTAAAC......CAGTCTGCTGGCCAAG3'
TCTGCTGGCCAAG GGGCCTTTACAACAACTA
chr11:92085261-92088570 FAT3
5' GATGGAAGTATGATGTGATGGA......TCAGTATAGACGACGAGAGTG 3'
AGTATAGACGACGAGAGTG GATGGAAGTATGATGTGAT

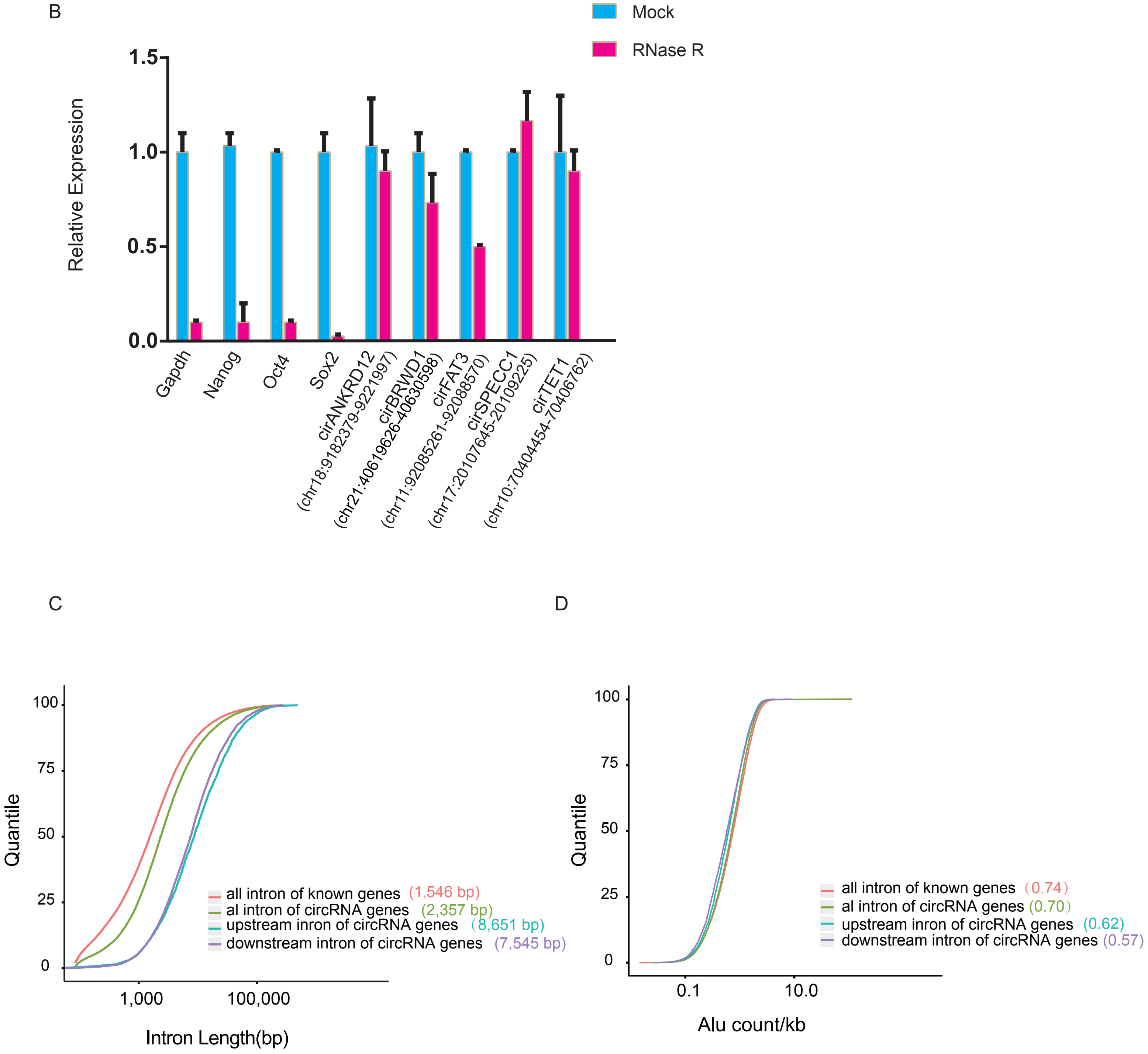

Figure S3. Feature analysis of the flanking introns of circRNAs.

(A) Validation of circRNA candidates in hESCs by Sanger seq. The back-spliced sies of these circRNAs are detected as a reversed order in the genome.

(B) Verification of circRNA candidates by RNase R digestion, The linear transcripts from four genes are degraded after RNase R treatment. Differently, these circRNA transcripts are resistant to the RNase treatment, proving their circularized characteristics.

(C) Length distribution of the flanking introns of circRNAs and all other introns. The upstream (blue line) and downstream (purple line) flanking introns are much longer than the control introns (green and red line). The median number is given in the bracket.

(D) Density of Alu elements in the flanking introns of circRNAs and all other introns. The Alu element count per kilobase in the flanking introns (upstream in blue and downstream in purple) is comparable with the randomly selected control introns regardless of their locations in circRNA host gene (in green) or not (in red). The median number is given in the bracket.

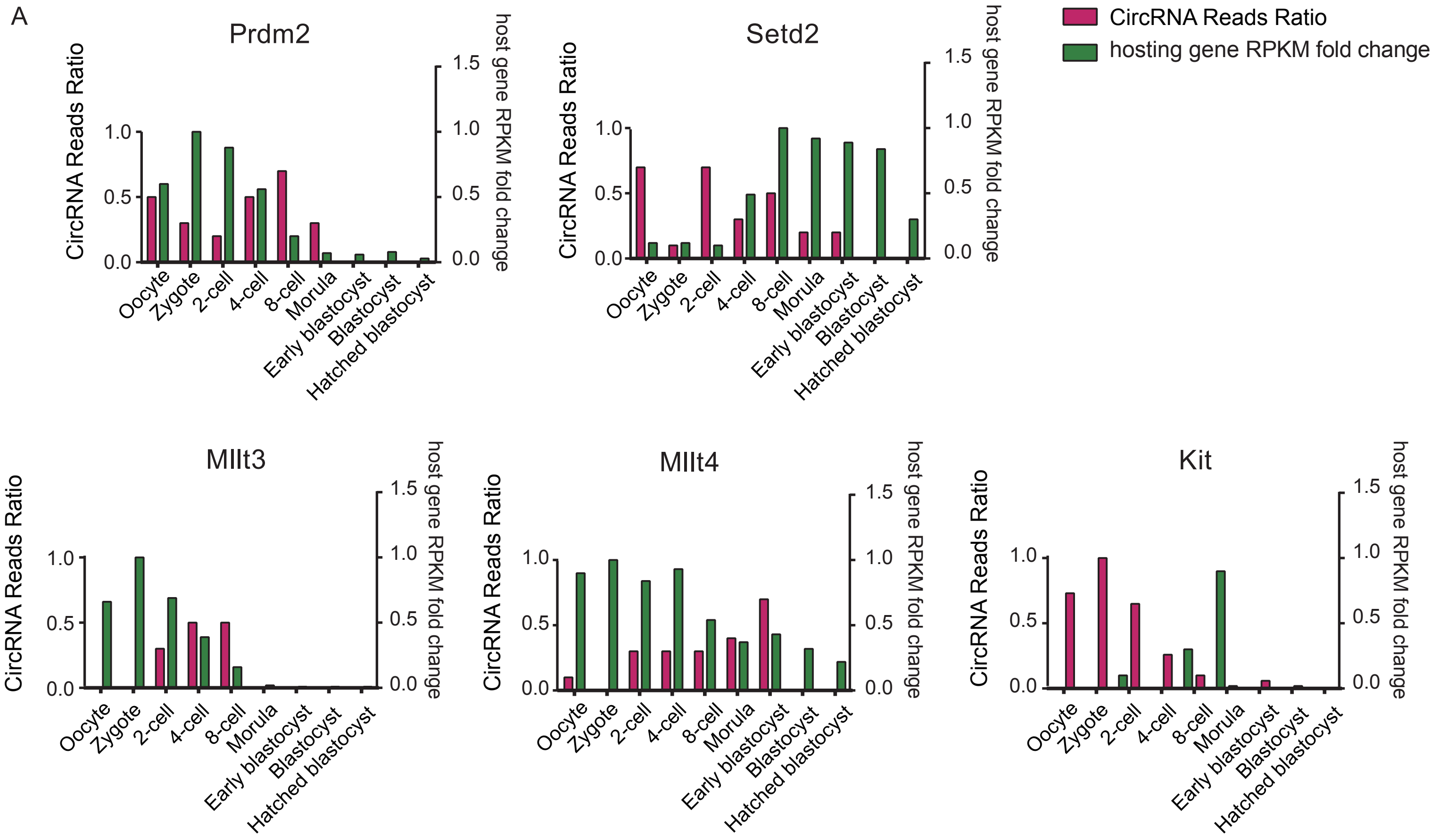
A
Prdm2
Setd2
Mllt3
Mllt4
Kit
CircRNA Reads Ratio
host gene RPKM fold change
CircRNA Reads Ratio
hosting gene RPKM fold change
Oocyte
Zygote
2-cell
4-cell
8-cell
Morula
Early blastocyst
Blastocyst
Hatched blastocyst

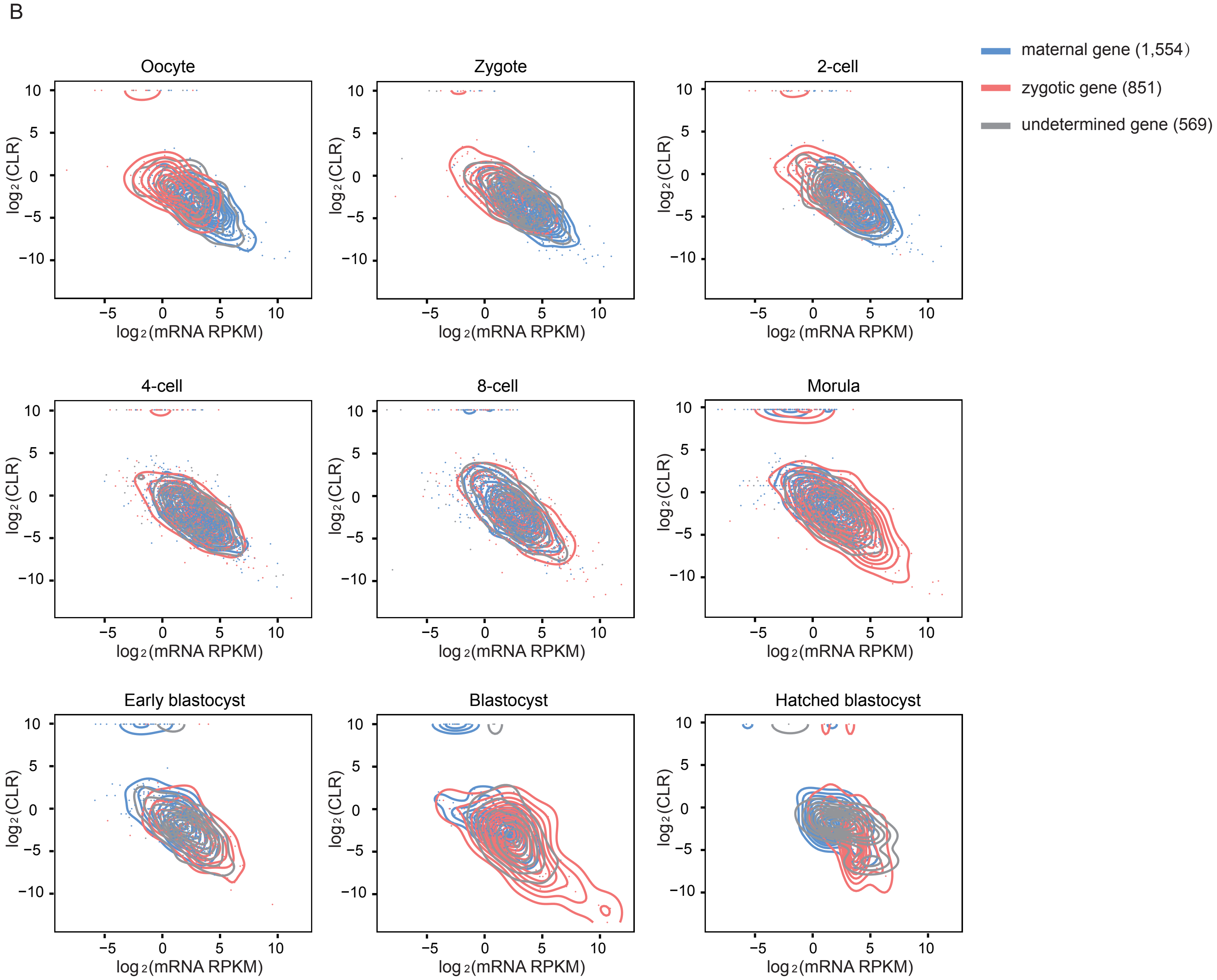
B
Oocyte
Zygote
2-cell
4-cell
8-cell
Morula
Early blastocyst
Blastocyst
Hatched blastocyst
log2(CLR)
log2(mRNA RPKM)
maternal gene (1,554)
zygotic gene (851)
undetermined gene (569)

C

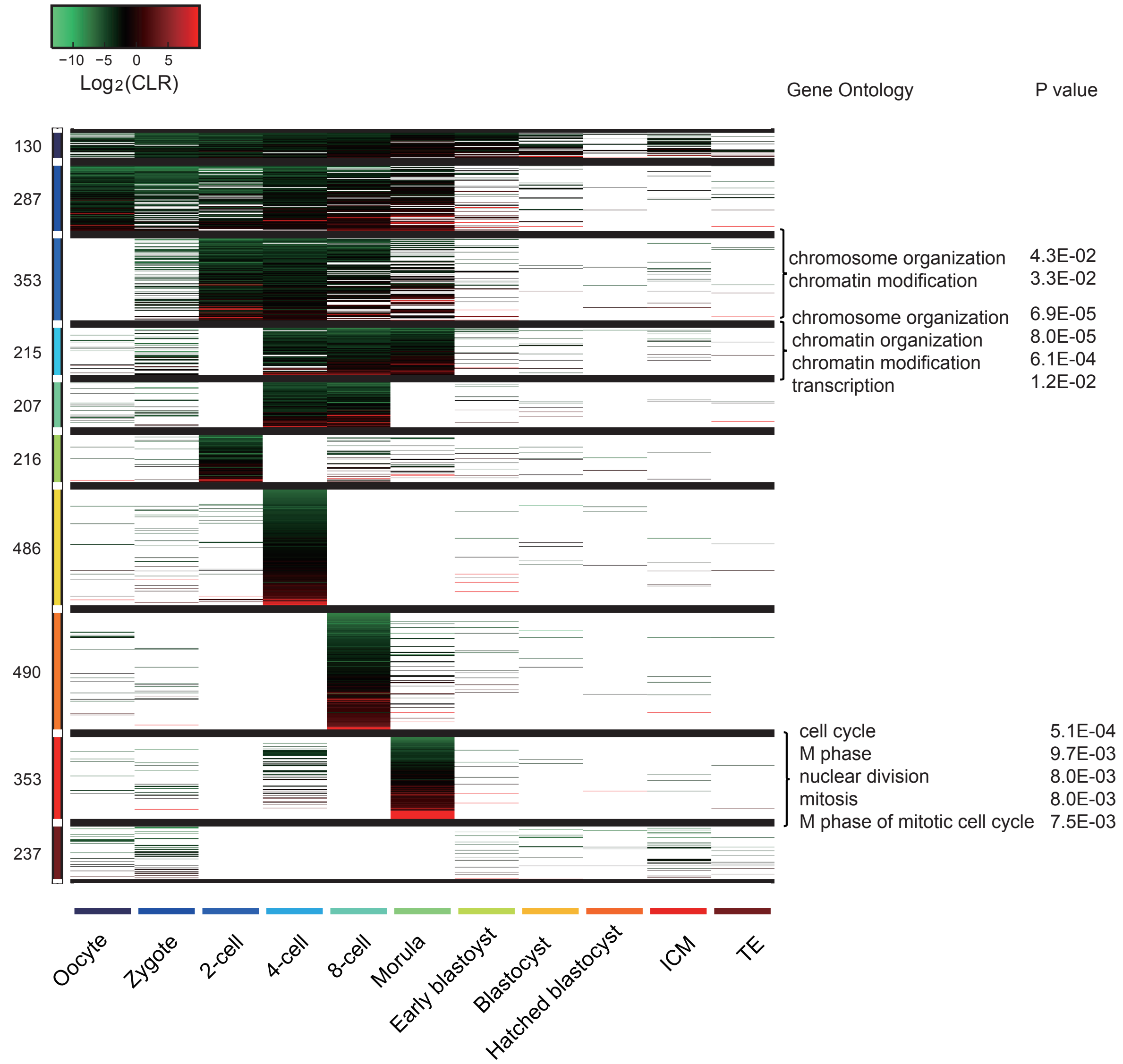
−10 −5 0 5
Log2(CLR)
Gene Ontology
P value
130
287
353
215
207
216
486
490
353
237
chromosome organization 4.3E-02
chromatin modification 3.3E-02
chromosome organization 6.9E-05
chromatin organization 8.0E-05
chromatin modification 6.1E-04
transcription 1.2E-02
cell cycle 5.1E-04
M phase 9.7E-03
nuclear division 8.0E-03
mitosis 8.0E-03
M phase of mitotic cell cycle 7.5E-03
Oocyte
Zygote
2-cell
4-cell
8-cell
Morula
Early blastoyst
Blastocyst
Hatched blastocyst
ICM
TE

D

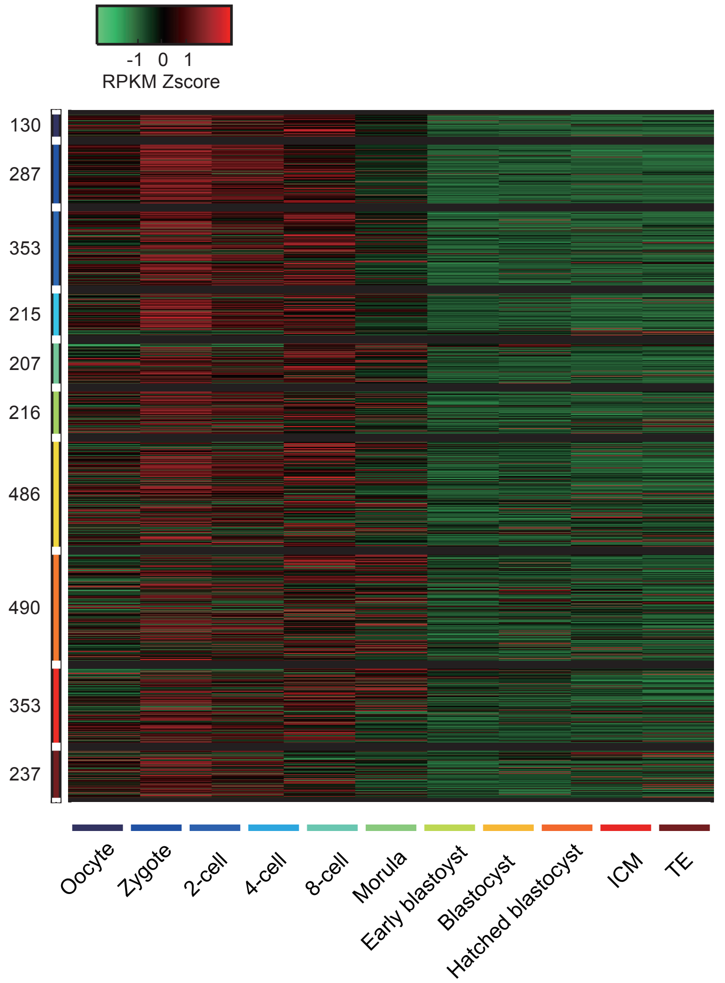
-1 0 1
RPKM Zscore
130
287
353
215
207
216
486
490
353
237
Oocyte
Zygote
2-cell
4-cell
8-cell
Morula
Early blastoyst
Blastocyst
Hatched blastocyst
ICM
TE

Figure S4. CircRNA and hosting gene expression patterns during human pre-implantation development.
(A) Dynamics of the circRNA expression level and corresponding hosting gene RPKM fold changes during pre-implantation. Five genes, as PRDM2, SETD2, MLLT3, MLLT4 and KIT, were selected because their circular reads ratio exceeded 0.5 as their circular transcripts' levels were higher than the level of the linear counterparts. Histograms show the circular reads ratio (in red) and the hosting gene RPKM fold-change normalized by the highest value (in green).
(B) Plot of CLR (circular to linear ratio) against the RPKM of the hosting gene showing the significant negative relationship between the abundance of circRNA and the hosting gene. The plotted hosting genes are clustered as zygotic genes (pink dots), maternal genes (blue dots) and undetermined genes (grey dots) according to the differential expressed gene list (Figure 2B).
(C) The clusters of circRNA hosting genes based on CLR value during human pre-implantation development. The enriched GO terms and corresponding P values are shown at right.
(D) The expression level of circRNA hosting genes. The genes are clustered according to Figure S4C.

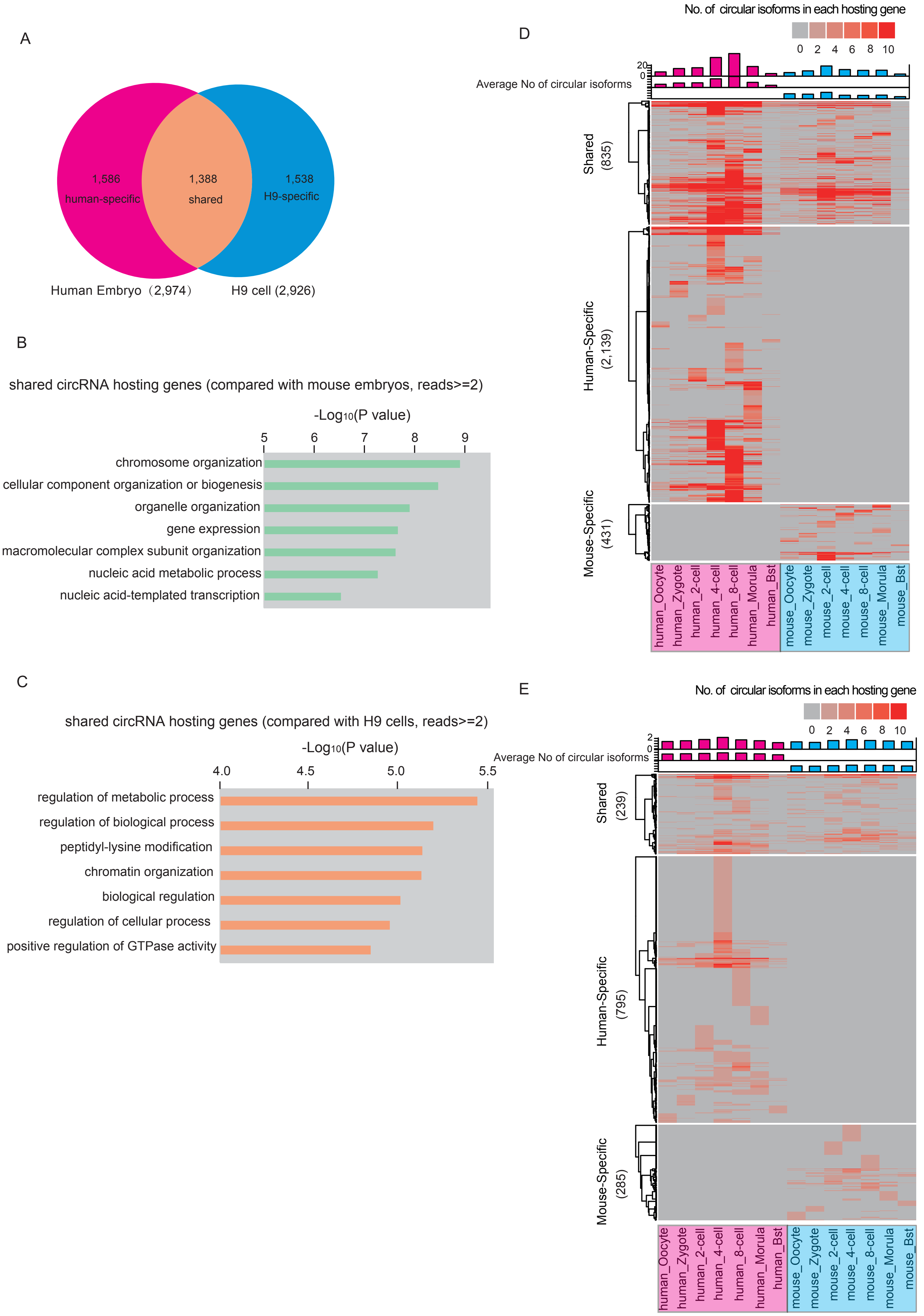
A
1,586
human-specific
1,388
shared
1,538
H9-specific
Human Embryo（2,974）
H9 cell (2,926)
B
shared circRNA hosting genes (compared with mouse embryos, reads>=2)
-Log10(P value)
5
6
7
8
9
chromosome organization
cellular component organization or biogenesis
organelle organization
gene expression
macromolecular complex subunit organization
nucleic acid metabolic process
nucleic acid-templated transcription
C
shared circRNA hosting genes (compared with H9 cells, reads>=2)
-Log10(P value)
4.0
4.5
5.0
5.5
regulation of metabolic process
regulation of biological process
peptidyl-lysine modification
chromatin organization
biological regulation
regulation of cellular process
positive regulation of GTPase activity
D
No. of circular isoforms in each hosting gene
0 2 4 6 8 10
Average No of circular isoforms
20
0
Shared (835)
Human-Specific (2,139)
Mouse-Specific (431)
human_Oocyte
human_Zygote
human_2-cell
human_4-cell
human_8-cell
human_Morula
human_Bst
mouse_Oocyte
mouse_Zygote
mouse_2-cell
mouse_4-cell
mouse_8-cell
mouse_Morula
mouse_Bst
E
No. of circular isoforms in each hosting gene
0 2 4 6 8 10
Average No of circular isoforms
2
0
Shared (239)
Human-Specific (795)
Mouse-Specific (285)
human_Oocyte
human_Zygote
human_2-cell
human_4-cell
human_8-cell
human_Morula
human_Bst
mouse_Oocyte
mouse_Zygote
mouse_2-cell
mouse_4-cell
mouse_8-cell
mouse_Morula
mouse_Bst

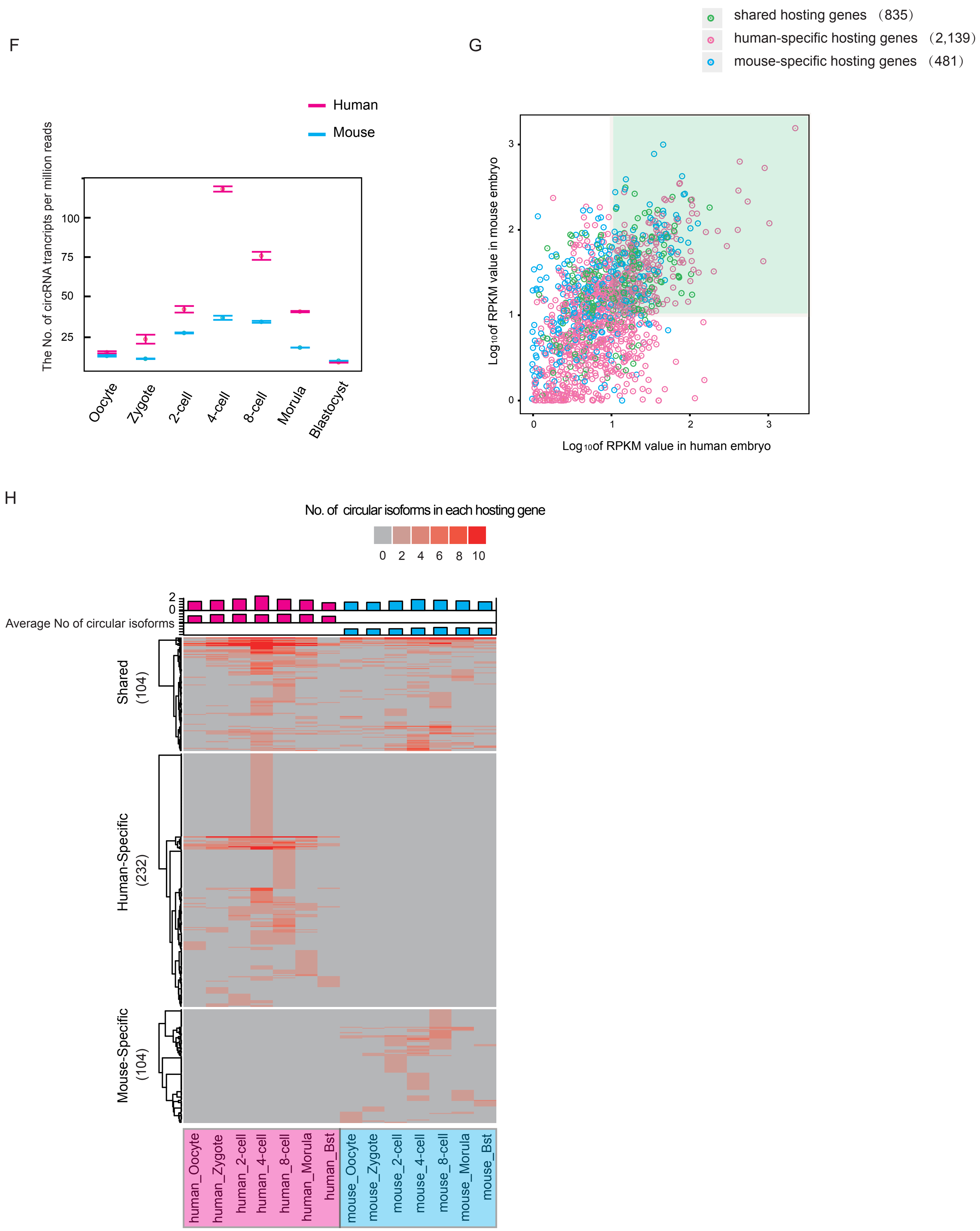
F
Human
Mouse
The No. of circRNA trancripts per million reads
100
75
50
25
Oocyte
Zygote
2-cell
4-cell
8-cell
Morula
Blastocyst
G
shared hosting genes (835)
human-specific hosting genes (2,139)
mouse-specific hosting genes (481)
Log10of RPKM value in mouse embryo
Log10of RPKM value in human embryo
0
1
2
3
H
No. of circular isoforms in each hosting gene
0 2 4 6 8 10
Average No of circular isoforms
Shared (104)
Human-Specific (232)
Mouse-Specific (104)
human_Oocyte
human_Zygote
human_2-cell
human_4-cell
human_8-cell
human_Morula
human_Bst
mouse_Oocyte
mouse_Zygote
mouse_2-cell
mouse_4-cell
mouse_8-cell
mouse_Morula
mouse_Bst

Figure S5. CircRNA hosting gene comparison.

(A) The Venn diagram shows that the majority of genes that express circRNAs in H9 cells also produce circular transcripts in human embryos.

(B) The top enriched gene ontology (GO) terms of the shared circRNA hosting genes between human embryos and mouse embryos are shown.

(C) The top enriched gene ontology (GO) terms of the shared circRNA hosting genes between human embryos and H9 cells are shown.

(D) Heat map of circularized level of all circRNA hosting genes during human and mouse pre-implantation development. The shared, human-specific and mouse-specific hosting genes are corresponding to the three parts of hosting genes from the Venn diagram in Figure 5A.

(E) Heat map of circularized level of circRNA hosting genes during human and mouse pre-implantation development after sub-sample the sequencing data.

(F) The box plots show the numbers of circular transcripts in human embryos (in red) and mouse embryos (in blue), during pre-implantation development, and the number was normalized to the total mapped reads (in millions) after subsampling the sequencing data.

(G) The scatter plot shows the distribution of the three kinds of circRNA hosting genes according to their expression level in human and mouse embryos. The values of log10 of (max RPKM during pre-implantation) are shown. And the highly expressed hosting genes are outlined as the RPKM >10, simultaneously in human and mouse embryos.

(H) Heat map of circularized level of circRNA hosting genes which are highly expressed during human and mouse pre-implantation development after subsampling the sequencing data.

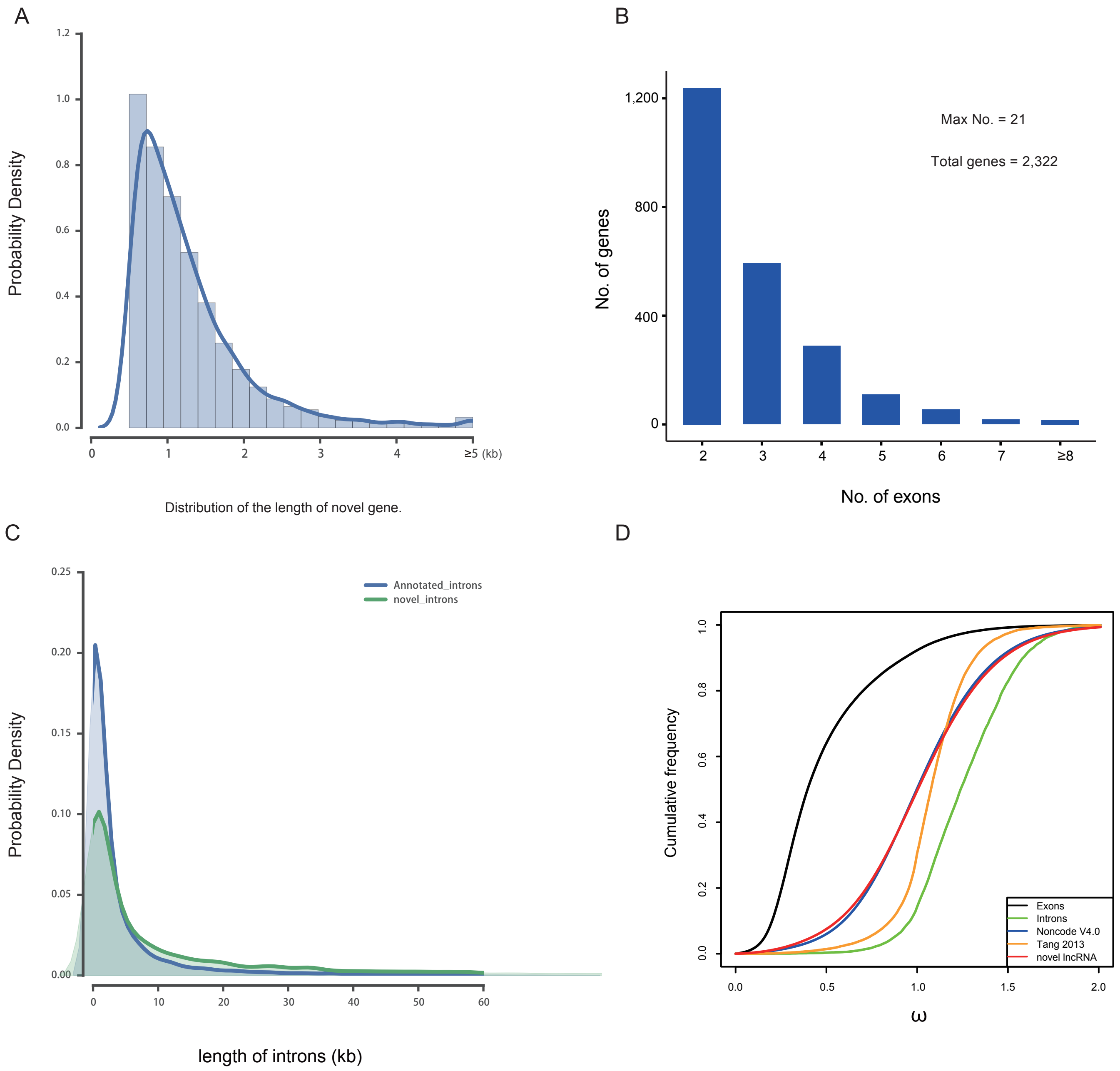

Figure S6. De novo assembled transcripts in the human pre-implantation embryos.
(A) Length distribution of the novel transcripts; candidates with lengths less than 500 bp are excluded.
(B) Exon number distribution of 2,322 novel genes; candidates with lengths less than 500 bp are excluded.
(C) The intron length distribution of the novel transcripts. The introns of novel genes are slightly longer than control introns of annotated genes.
(D) Conservation level (ω metric) of the novel transcripts compared with exons, introns, Noncode V4.0 database and novel lncRNAs identified previously, indicating that novel transcripts are potential lncRNAs.
