# Supplementary material for: Tracing the expression of circular RNAs in human pre-implantation embryos
Source: Genome Biol. 2016 Jun 17;17:130. doi: 10.1186/s13059-016-0991-3 (PMC4911693; doi:10.1186/s13059-016-0991-3)
Supplement: Additional file 8: Table S7. — Sequences of RGC-A80 and the primers used in ddPCR and circRNAs validation. The sequences of RGC-A80, the primers used in ddPCR for detecting the copy number of 44 genes, and the primers of circRNAs validation are listed. (DOCX 19 kb) [file 13059_2016_991_MOESM8_ESM.docx]

| RGC-A80 | sequence |
| --- | --- |
| RFP_812 | ACACTATAGAATACAAGCTACTTGTTCTTTTTGCAGGATCCCGCCACCATGGCCTCCTCCGAGGACGTCATCAAGGAGTTCATGCGCTTCAAGGTGCGCATGGAGGGCTCCGTGAACGGCCACGAGTTCGAGATCGAGGGCGAGGGCGAGGGCCGCCCCTACGAGGGCACCCAGACCGCCAAGCTGAAGGTGACCAAGGGCGGCCCCCTGCCCTTCGCCTGGGACATCCTGTCCCCTCAGTTCCAGTACGGCTCCAAGGCCTACGTGAAGCACCCCGCCGACATCCCCGACTACTTGAAGCTGTCCTTCCCCGAGGGCTTCAAGTGGGAGCGCGTGATGAACTTCGAGGACGGCGGCGTGGTGACCGTGACCCAGGACTCCTCCCTGCAGGACGGCGAGTTCATCTACAAGGTGAAGCTGCGCGGCACCAACTTCCCCTCCGACGGCCCCGTAATGCAGAAGAAGACCATGGGCTGGGAGGCCTCCACCGAGCGGATGTACCCCGAGGACGGCGCCCTGAAGGGCGAGATCAAGATGAGGCTGAAGCTGAAGGACGGCGGCCACTACGACGCCGAGGTCAAGACCACCTACATGGCCAAGAAGCCCGTGCAGCTGCCCGGCGCCTACAAGACCGACATCAAGCTGGACATCACCTCCCACAACGAGGACTACACCATCGTGGAACAGTACGAGCGCGCCGAGGGCCGCCACTCCACCGGCGCCTAACTCGAGAAAAAAAAAAAAAAAAAAAAAAAAAAAAAAAAAAAAAAAAAAAAAAAAAAAAAAAAAAAAAAAAAAAAAAAAAAAAAAAA |
| GFP_866 | ACACTATAGAATACAAGCTACTTGTTCTTTTTGCAGGATCCAATCGCCGGTCGCCACCATGGTGAGCAAGGGCGAGGAGCTGTTCACCGGGGTGGTGCCCATCCTGGTCGAGCTGGACGGCGACGTAAACGGCCACAAGTTCAGCGTGTCCGGCGAGGGCGAGGGCGATGCCACCTACGGCAAGCTGACCCTGAAGTTCATCTGCACCACCGGCAAGCTGCCCGTGCCCTGGCCCACCCTCGTGACCACCCTGACCTACGGCGTGCAGTGCTTCAGCCGCTACCCCGACCACATGAAGCAGCACGACTTCTTCAAGTCCGCCATGCCCGAAGGCTACGTCCAGGAGCGCACCATCTTCTTCAAGGACGACGGCAACTACAAGACCCGCGCCGAGGTGAAGTTCGAGGGCGACACCCTGGTGAACCGCATCGAGCTGAAGGGCATCGACTTCAAGGAGGACGGCAACATCCTGGGGCACAAGCTGGAGTACAACTACAACAGCCACAACGTCTATATCATGGCCGACAAGCAGAAGAACGGCATCAAGGTGAACTTCAAGATCCGCCACAACATCGAGGACGGCAGCGTGCAGCTCGCCGACCACTACCAGCAGAACACCCCCATCGGCGACGGCCCCGTGCTGCTGCCCGACAACCACTACCTGAGCACCCAGTCCGCCCTGAGCAAAGACCCCAACGAGAAGCGCGATCACATGGTCCTGCTGGAGTTCGTGACCGCCGCCGGGATCACTCTCGGCATGGACGAGCTGTACAAGTAAAGCTCGAGAAAAAAAAAAAAAAAAAAAAAAAAAAAAAAAAAAAAAAAAAAAAAAAAAAAAAAAAAAAAAAAAAAAAAAAAAAAAAAAA |
| Cre_1,186 | ACACTATAGAATACAAGCTACTTGTTCTTTTTGCAGGATCAGNTATGCCCAAGAAGAAGAGGAAGGTGTCCAATTTACTGACCGTACACCAAAATTTGCCTGCATTACCGGTCGATGCAACGAGTGATGAGGTTCGCAAGAACCTGATGGACATGTTCAGGGATCGCCAGGCGTTTTCTGAGCATACCTGGAAAATGCTTCTGTCCGTTTGCCGGTCGTGGGCGGCATGGTGCAAGTTGAATAACCGGAAATGGTTTCCCGCAGAACCTGAAGATGTTCGCGATTATCTTCTATATCTTCAGGCGCGCGGTCTGGCAGTAAAAACTATCCAGCAACATTTGGGCCAGCTAAACATGCTTCATCGTCGGTCCGGGCTGCCACGACCAAGTGACAGCAATGCTGTTTCACTGGTTATGCGGCGGATCCGAAAAGAAAACGTTGATGCCGGTGAACGTGCAAAACAGGCTCTAGCGTTCGAACGCACTGATTTCGACCAGGTTCGTTCACTCATGGAAAATAGCGATCGCTGCCAGGATATACGTAATCTGGCATTTCTGGGGATTGCTTATAACACCCTGTTACGTATAGCCGAAATTGCCAGGATCAGGGTTAAAGATATCTCACGTACTGACGGTGGGAGAATGTTAATCCATATTGGCAGAACGAAAACGCTGGTTAGCACCGCAGGTGTAGAGAAGGCACTTAGCCTGGGGGTAACTAAACTGGTCGAGCGATGGATTTCCGTCTCTGGTGTAGCTGATGATCCGAATAACTACCTGTTTTGCCGGGTCAGAAAAAATGGTGTTGCCGCGCCATCTGCCACCAGCCAGCTATCAACTCGCGCCCTGGAAGGGATTTTTGAAGCAACTCATCGATTGATTTACGGCGCTAAGGATGACTCTGGTCAGAGATACCTGGCCTGGTCTGGACACAGTGCCCGTGTCGGAGCCGCGCGAGATATGGCCCGCGCTGGAGTTTCAATACCGGAGATCATGCAAGCTGGTGGCTGGACCAATGTAAATATTGTCATGAACTATATCCGTAACCTGGATAGTGAAACAGGGGCAATGGTGCGCCTGCTGGAAGATGGCGATTAGGGTCTCGAGAAAAAAAAAAAAAAAAAAAAAAAAAAAAAAAAAAAAAAAAAAAAAAAAAAAAAAAAAAAAAAAAAAAAAAAAAAAAAAAA |

| **Primers used in ddPCR experiments** | |
| --- | --- |
| AGO4_F | GGCAGGACAGCGATGTATCA |
| AGO4_R | TCCACCCACCATACTGTTGC |
| CBX1_F | GACCGTCGAGTGGTAAAGGG |
| CBX1_R | AACTCAGCAATGAGGTCGGG |
| CD46_F | TTGCCATAGGAAAGCAGATGGT |
| CD46_R | GCTCTGCTGGAGTGGTTGAT |
| CDK1_F | CTGGGGTCAGCTCGTTACTC |
| CDK1_R | TCCACTTCTGGCCACACTTC |
| CDK7_F | ACTTTGGGCACACCAACTGA |
| CDK7_R | TGGCCGTAATTCGAGCACAT |
| CDR1_F | CTGGAGGCCATTGGAAGATGT |
| CDR1_R | AAGTCTTCCGGATAATTTGGGTCT |
| CNOT1_F | ACGCAGACGTCAGTGGAAAT |
| CNOT1_R | GATGGGAGAGGAGGAGGTGT |
| CNOT11_F | CTCTCCCTGAGTGTCGAGGA |
| CNOT11_R | AATGCTTTGCGGAGGAGACA |
| CNOT7-F | CGAGCTCAGCGACACAAGTA |
| CNOT7-R | TGGCATAGTGAGGGCACAAG |
| CNOT8-F | TGAGCGAGCGCAACTAATCA |
| CNOT8-R | AGCAGAAGTAACAGGGTGCC |
| CUL3_F | CCCCATGCGTCTTGGGTTAT |
| CUL3_R | TGTAGAGATAAAGAGCACAAGGCT |
| DCP2_F | AGATGGTGGGCTTTCTCAGC |
| DCP2_R | TTGAAAGCATGGGGCAAAGC |
| DEK_F | GAAAGAACCCGAAATGCCCG |
| DEK_R | AGTGCCTGGCCTGTTGTAAA |
| DHX9_F | GCCAATTTCTGGCCAAAGCA |
| DHX9_R | CGAGGCTCAATGGGGAGTTT |
| DHX15_F | GACCCAAGAGAGGAGTTGCC |
| DHX15_R | GCAACTCTCTGAGCCACACT |
| DNMT1-F | CATGAGTGCATTGGTGGCTG |
| DNMT1-R | CTTCCACGCAGGAGCAGAC |
| DNMT3B-F | GGAGAGAGCAAACAAAGGGGT |
| DNMT3B-R | GGCCCCTTCAAACAATGCC |
| DPP4_F | TTTAACGACACAGAAGTCCCACT |
| DPP4_R | TTGGATATGGAACCCGTACAGTC |
| DPPA2_F | GTGTGGTCCATGGCAGACTT |
| DPPA2_R | CTCCTGTGAGTGGTAGGCAC |
| DPPA3-F | GCAGCAGTCCTCAGGGAAAT |
| DPPA3-R | CGAACTCCGCCGAGTAACAT |
| DPPA5-F | GTAGACATATCCCGCCGTGG |
| DPPA5-R | AAAATGGCTTTCAGCAGCCG |
| EIF3M_F | TCATGGTGGAATTGCTCGGAA |
| EIF3M_R | ACACCTGTGGGCATCAACTC |
| HOMER1_F | CTCTCCCTGAGTGTCGAGGA |
| HOMER1_R | AATGCTTTGCGGAGGAGACA |
| MYC_F | CACGTCTCCACACATCAGCA |
| MYC_R | TGTGTGTTCGCCTCTTGACA |
| NANOG-F | CAGAAGGCCTCAGCACCTAC |
| NANOG-R | ATTGTTCCAGGTCTGGTTGC |
| PAIP2_F | TTTCCACACTGTGAAGGCAGT |
| PAIP2_R | AGCCATGGGGCCAATTCTATT |
| PDIA4_F | CTCCACCAGAAGTCACGCTT |
| PDIA4_R | GGGGCAAGTTTCTTGCAGTG |
| PDIA6_F | TGAGCAAGGCATCAACGAGT |
| PDIA6_R | CCCAAGGCTCTCTCTCAACG |
| PKD2_F | CTGCATCGCCACCTCCC |
| PKD2_R | CTTGTTCCCCAGAGACCTCG |
| POLR2D-F | AGCCCATAGCCCCTTGAGAT |
| POLR2D-R | CTCTTTGGCACGACCATTGC |
| POU5F1-F | AGTGCCCGAAACCCACAC |
| POU5F1-R | GGAGACCCAGCAGCCTCAAA |
| RPLP0-F | CCTCGTGGAAGTGACATCGT |
| RPLP0-R | ATCTGCTTGGAGCCCACATT |
| SIRT1_F | GAGCTGGGGTGTCTGTTTCA |
| SIRT1_R | GGAAGTCTACAGCAAGGCGA |
| SOX12_F | GAAGCAAGATTGGGCCACAC |
| SOX12_R | TGGAGTAACACGATGGGCAC |
| SOX2-F | AACCAGCGCATGGACAGTTA |
| SOX2-R | GACTTGACCACCGAACCCAT |
| TCEB1-F | TGCATGGGGAAAGAAGCACT |
| TCEB1-R | GCCAGTCTCAAATAAGCTGGAG |
| TET1-F | ACAGGCCTTTGGTGCTATCC |
| TET1-R | CTGGGACAACACTCCCACTC |
| TET2-F | GAGGCTAGGCTGCTTTCGTA |
| TET2-R | ATGTTTGCCAGCCTCGTTCT |
| TET3_F | CAATGGTGCTAGAGAGCCCG |
| TET3_R | TAAGAGGACACAGCTTCGGC |
| TPI1_F | GGCATGATCAAAGACTGCGG |
| TPI1_R | ACCTTCTCAGTGATGCCAGC |
| UGP2-F | GCTCAAGGTGTGCATGTGTG |
| UGP2-R | TAGCAGCTGGGAAATTGGGG |
| USB1_F | GAGTCATGGTGCGGGAATGA |
| USB1_R | GGCTTTTGTGCAACACCTCA |
| YAP1_F | CCACAGGCAATGCGGAATATC |
| YAP1_R | GGATCTGAGCTATTGGTCGTCA |
| ZNRD1_F | GTGCGTGGCAGGAGGAC |
| ZNRD1_R | ACAGCGAATACAGGTGACCG |
| **Primers of circRNA candidates** | |
| ANKRD12- cir-F | TAAACATGGGGAGCGTCCAG |
| ANKRD12-cir-R | TCTTCTCATCCTGGATCTGTGTAAC |
| BRWD1-cir-F | CAAGCTCTGTAAGACATGTGATGG |
| BRWD1-cir-R | CTCCGGGGTGTTTCTTCACC |
| FAT3-cir-F | CGGAAGGGATGGAGAGATCC |
| FAT3-cir-R | TCTGGCTGTTGACGTAGGTC |
| SPECC1-cir-F | TGTTGAAAGTAGCCCGAGCA |
| SPECC1-cir-R | GATTTCCTGGGGTTGGAGGG |
| TET1-cir-F | ATTCTGAACTGCCCACCTGC |
| TET1-cir-R | TGTCCACTTCTCCACCTCGA |
